# Transformative potential of digital systems for promoting human-wildlife coexistence: A systematic literature review

**DOI:** 10.1007/s13280-026-02399-7

**Published:** 2026-05-21

**Authors:** Bas S. H. T. Michielsen, Gerard Schouten, Joris P. G. Cromsigt, Remco C. Veltkamp, Irma Arts, Jitse Bijlmakers, Magali Frauendorf, Tim R. Hofmeester, Amy Newsom, Minh-Xuân A. Truong, Helen Verploegen, Els Weinans, Wenjin Yu, Silja Zimmermann, Ine Dorresteijn

**Affiliations:** 1 Copernicus Institute of Sustainable Development, Utrecht University, Utrecht, The Netherlands; 2 School of ICT, Fontys University of Applied Sciences, Eindhoven, The Netherlands; 3 Information and Computing Sciences, Utrecht University, Utrecht, The Netherlands; 4 Department of Wildlife, Fish, and Environmental Studies, Swedish University of Agricultural Sciences, Umeå, Sweden; 5 Centre for African Conservation Ecology, DepartmentofZoology, Nelson Mandela University, Gqeberha, South Africa; 6 Ecology Department, Swedish University of Agricultural Sciences, Uppsala, Sweden; 7 Institute for Science in Society, Radboud University, Nijmegen, The Netherlands; 8 Environmental Sciences, Open Universiteit, Heerlen, The Netherlands

**Keywords:** Digital technology, Human-wildlife interaction, Sustainable development, Transformative potential, Wildlife management and conservation

## Abstract

Digital technology plays an increasingly important role in wildlife management and conservation, by enhancing monitoring capabilities and reshaping human-wildlife interactions. However, the transformative potential of these digital solutions for coexistence remains unclear. This paper presents a novel framework to assess the transformative potential of digital systems in wildlife management and conservation, focusing on two key factors: Digital Maturity, which evaluates technical sophistication of digital systems, and Systemic Depth, which measures their capacity for enabling lasting change. We used this framework to evaluate 524 studies in a systematic literature review in wildlife management and conservation, and found that although sometimes higher Digital Maturity or Systemic Depth was achieved, overall the transformative potential of applied digital systems was still low. Studies that scored high emphasize interdisciplinary collaboration, adaptability, data sharing, and technologies such as machine learning. This research highlights achieving transformative potential requires a holistic approach integrating ecological, social, and technological perspectives.

**Supplementary Information:**

The online version contains supplementary material available at https://doi.org/10.1007/s13280-026-02399-7.

## Introduction

In the field of wildlife management and conservation innovative digital solutions increasingly transform traditional practices and enhance the ability to monitor, conserve, and manage wildlife populations (Berger-Tal and Lahoz-Monfort 2018), a trend that mirrors the wider digital transformation in society. Examples include systems to detect poachers in various African countries (Edemacu et al. 2019); automated computer vision methods to detect animal intrusions and prevent human-animal conflict in India (Bhagabati et al. 2024); and drones to detect sharks and keep the beaches of Australia safe for humans (Butcher et al. 2019). Additionally, the recent comeback of wild animals has increased tensions in co-existence between humans and wildlife, for example wolves and lynxes in Western Europe (Enserink and Vogel 2006; Davoli et al*.* 2022). In many of these cases digital technologies are looked upon to provide solutions (Berger-Tal and Lahoz-Monfort 2018; von Essen et al. 2023).

In wildlife management and conservation, digital systems are used for different purposes and address issues on varying levels of society. Some systems are implemented primarily to monitor wildlife and support population density estimates for instance, for conservation (Murphy et al. 2019; McCarthy et al. 2022) or management purposes (von Essen et al. 2023), whereas other systems aim to influence animal or human behaviour with the goal of avoiding human-animal conflict (Dertien et al. 2023) or improving co-existence (Panda et al. 2022). Additionally, digital technologies have been used to raise awareness, support policymakers and/or change the general population’s view on wild animals and wildlife management (Kobayashi and Matsushima 2014).

In general, when implemented thoughtfully, addressing questions of data ownership and control (Adams 2019), digital systems have the potential to yield significant transformational benefits; conversely, if digitalization is approached reductively, it may lead to the creation of technological fixes that fail to address the underlying issues they were intended to resolve, resulting in low transformational potential (Schneider and Kokshagina 2021). However, a coherent framework to assess the potential of digital systems to evoke transformative change in human-wildlife interactions is lacking to date, especially for evaluating their transformative potential.

Here, we present a new framework to examine the transformative potential of digital systems, specifically to assess how they can support human-wildlife coexistence and promote sustainable practices. Our framework is founded on two independent factors, that each drive transformative potential in a different way. The first factor is related to the intrinsic technical characteristics of the digital system. If the digital system in question merely collects and visualizes data, it has a lower potential to achieve transformative impact in practice than a system that also shares collected data with other systems or uses these data to create a predictive model to generate data-informed recommendations. This perspective is consistent with findings in the literature (Gökalp and Martinez 2021), which evaluated digital transformation capability in business innovation. In this review, we operationalized this factor as **Digital Maturity**. The second factor stems from sustainability sciences and evaluates the potential of the digital system to transform current practices into a long-lasting, more sustainable solution. It is inspired by the leverage points framework (Meadows 1999) and its system characteristics (Abson et al. 2017). In this review, we operationalized this factor as **Systemic Depth**.

In this review paper, our aim is to evaluate the transformative potential of digital systems as applied in wildlife management and conservation studies. We first describe our theoretical framework for assessing the transformative potential of digital systems in sustainability transformations in general. Second, we present a systemic literature review to (1) examine the use of digital systems in wildlife management and conservation studies; (2) apply our framework to understand how the reviewed literature compares in terms of transformative potential; and (3) describe the traits of studies that the framework has identified as having high transformative potential.

## Theoretical framework

Here we will elaborate on the proposed framework, which is grounded in two key factors, Digital Maturity and Systemic Depth, that together define the transformative potential of digital systems in supporting sustainable practices in wildlife management and conservation. Building on the IPBES definition of transformative change (IPBES 2019) we understand the transformative potential of digital systems as their capacity to contribute to fundamental, scalable, system-wide reorganization of human-wildlife relations, including non-incremental changes to practices, institutions and underlying goals and values (Feola 2015; Arponen and Salomaa 2023). Additionally, we understand sustainable practices in wildlife management as those that maintain long-term wildlife populations and habitats while also supporting socially legitimate, equitable, and adaptive human-wildlife relations (Carter and Linnell 2023), managing trade-offs, tolerable risk, and long-term coexistence rather than zero-conflict ideals (Thapa et al. 2024).

Digitalization is a fundamental driver of large-scale transitions across diverse sectors such as healthcare, business, and wildlife management. For example, in healthcare, AI-powered diagnostic systems revolutionize patient care by enabling more personalized and timely treatments (Khalifa and Albadawy 2024), and in business, machine learning tools enhance transparency and improve risk assessment (Batz et al. 2025). Similarly, the process of digitalization in wildlife management and conservation changes the field fundamentally, for example, the introduction of machine learning models like MegaDetector (Beery et al. 2019) and SpeciesNet (Gadot et al. 2024) that detect and identify animals in camera trap images in semi-real-time, avoiding the need for wildlife managers to go through these data manually, significantly increasing the timeliness and adaptability of management practices. However, we realize that overreliance on digitalization and digital systems without care for established principles for socially responsible use (Sandbrook et al. 2021), for instance through data-driven management frameworks, algorithmic monitoring, or increasingly pervasive surveillance technologies, can redistribute power and marginalize local stakeholders, introducing new ethical and governance challenges (Arts et al. 2015; Adams 2019; Simlai and Sandbrook 2021).

Progression in digitalization is typically measured using Digital Maturity assessments, which provide degrees of development in aspects like data visualization, modelling capacity, data-driven decision making, and data storage and sharing. Different assessments exists that agree on defining maturity as recognizing digital transformations as multidimensional shifts that exceeds technology adoption and require a clear, precise strategy to align organizational efforts with its digital focus (Ochoa-Urrego and Peña-Reyes 2021).

To define a metric for digitalization, we adopted and extended the characteristics from the Three Pillars of Data Analytics (Raghupathi and Raghupathi 2021) and termed it Digital Maturity. It is worthwhile to note that, though useful, the descriptions of these pillars are not conceptually neutral: visualizations involves selective storytelling, statistical modelling depends on simplifying assumptions about cleanness of data and structure, and data-driven patterns in machine learning can reflect biases embedded in historical data. Since the pillars lack indicators for data management, we introduced a fourth characteristic focused on how the study stores, shares, and integrates data, based on the FAIR Guiding Principles (Wilkinson et al. 2016). These principles emphasize that data should be Findable, Accessible, Interoperable, and Reusable to enhance its usability and facilitate data sharing and collaboration. Our characteristic ranges from sharing data via a simple download link, which is a valid method but likely disregards the FAIR principles, to storing and sharing data in a FAIR-compliant manner, possibly including data maintenance plans and explicitly describing data integration to facilitate reuse. As such, the Digital Maturity score is defined by the characteristics *Visualizations*, *Statistical Modelling*, *Machine Learning* and *Data Storing/Sharing* (See Table 1 for definitions).

**Table 1:** The digitalization characteristics as described by Raghupathi and Raghupathi (2021), extended with data storing/sharing inspired by the FAIR Guiding Principes (Wilkinson et al. 2016)

Digitalization characteristics (Raghupathi and Raghupathi 2021) + Data storing/sharing	
Visualizations	Visual storytelling the data, including the various variables, dimensions, correlations and gained insights
Statistical modelling	Modelling based on strict rules and a set of assumptions using clean and clear data
Machine learning	Data driven problem solving by identifying patterns in unstructured and/or noisy data
Data storing/sharing	The status of efforts that relate to data collection, maintenance, and integration

Sustainable transformations in society do not just require incremental changes but transformative shifts in system functions (Scoones et al. 2020; West et al. 2020; Horcea-Milcu et al. 2024). Sustainability transformations involve profound systemic changes that fundamentally reshape the goals, structures, feedbacks, and parameters of complex systems to achieve sustainable outcomes (Meadows 1999; Abson et al. 2017).

The leverage points framework (Meadows 1999) provides an analytical framework for identifying places within complex systems where targeted interventions can lead to transformative change, and has been applied across diverse systems, ranging from smallholder farming (Manlosa et al. 2019; Fischer et al. 2022) to food and energy systems (Dorninger et al. 2020), marine and coastal pollution (Riechers et al. 2021), and indigenous and local knowledge systems (Burgos-Ayala et al. 2020; Zimmermann et al. 2023). More recently, Meadows’ leverage points framework was aggregated into four system characteristics, namely *Parameters*, *Feedbacks*, *Design*, and *Intent* (see Table 2 for definitions) (Abson et al. 2017).

**Table 2:** The system characteristics as defined by Abson et al. (2017), which we use as indicators for the level of systemic depth

System characteristics (Abson et al. 2017)	
Parameters	The relatively mechanistic characteristics typically targeted by policy makers
Feedbacks	The interactions between elements within a system of interest, that drive internal dynamics
Design	The social structures and institutions that manage feedbacks and parameters
Intent	The underpinning values, goals, and world views of actors that shape the emergent direction to which a system is oriented

To define a metric for sustainable transformations, we adopted these four system characteristics and termed them Systemic Depth. Lower levels of systemic depth, namely *Parameters* and *Feedbacks*, align with shallow leverage points. These points use an atomized focus to intervene at an elemental level within the system and are hypothesized to have limited capacity to induce transformative change (Meadows 1999; Abson et al. 2017). In contrast, higher levels of systemic depth, namely *Design* and *Intent*, correspond to deep leverage points which are hypothesized to have greater potential to induce transformative change (Meadows 1999; Abson et al. 2017). However, the framework is hierarchical, nested, and constraining, meaning that changes at one level may inhibit or accelerate changes at other levels. It is the interplay between shallow and deep levels that holds the greatest potential for transformative change (Fischer et al. 2022; Zimmermann et al. 2025). Thus, the leverage points perspective offers a valuable lens through which to assess the transformative potential of digital systems in facilitating societal transitions (Fischer and Riechers 2019).

**Fig. 1:**
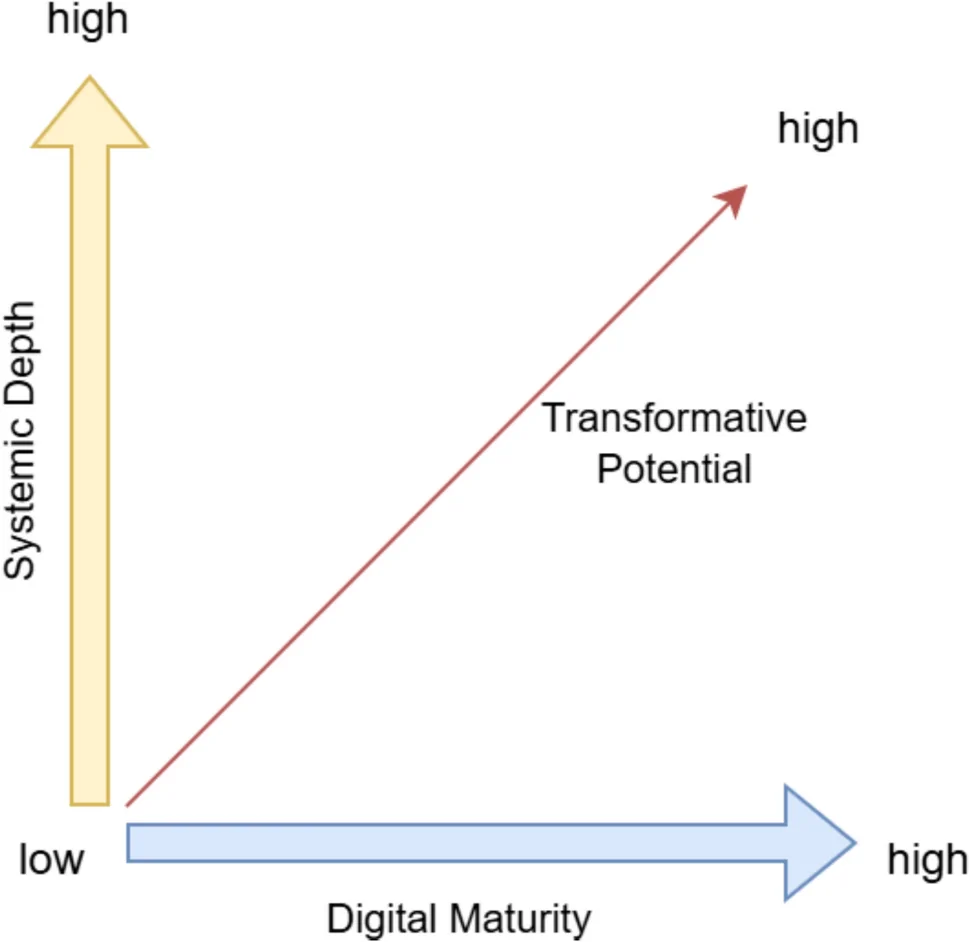
Schematic overview of the Digital Sustainability Transformation Framework (DSTF), a 2D model that assesses the transformative potential of projects utilizing digital systems. The axes represent metrics for Digital Maturity and Systemic Depth. Initiatives in the top left corner exhibit deep transformative capabilities but lack optimized digital solutions, while those in the bottom right corner utilize advanced digital technologies without leveraging full systemic depth. Projects positioned in the top right corner combine deep-reaching solutions with advanced digital technologies, indicating high transformative potential

Combining these metrics for digitalization (Digital Maturity) and sustainability transformations (Systemic Depth), we propose the Digital Sustainability Transformation Framework (DSTF). The DSTF is a 2D framework that combines scores on the two metrics to assess the transformative potential of projects applying digital systems. Our framework categorizes initiatives based on their capability to incorporate technological support to leverage change (Fig. 1). The top right corner in Fig. 1 represents projects that effectively integrate sustainable solutions with advanced digital technologies, signifying high transformative potential.

## Materials and methods

We followed the PRISMA methodology for systematic literature reviews, as outlined in the original PRISMA statement (Liberati et al. 2009) and further updated in the PRISMA 2020 statement (Page et al. 2021). Additionally, the screening process was significantly enhanced by ASReview v1.5 (van de Schoot et al. 2021), an active learning application that improves the efficiency of time spent by researchers in identifying relevant studies from search results.

### Data collection

On 2023-11-13, we searched both Scopus and Web of Science for studies using logically identical queries, applying no other filters.

Scopus (6351 records):

 TITLE-ABS-KEY ((wildlife OR “wild animal”) AND (technolog* OR sensor* OR robotic* OR device* OR (digital W/1 system*) OR (digital W/1 tool*)))

Web of Science (3066 records):

 (TI = (wildlife OR “wild animal”) OR AB = (wildlife OR “wild animal”) OR AK = (wildlife OR “wild animal”)) AND (TI = (technolog* OR sensor* OR robotic* OR devic* OR digital NEAR/1 system* OR digital NEAR/1 tool*) OR AB = (technolog* OR sensor* OR robotic* OR devic* OR digital NEAR/1 system* OR digital NEAR/1 tool*) OR AK = (technolog* OR sensor* OR robotic* OR devic* OR digital NEAR/1 system* OR digital NEAR/1 tool*))

Duplicate results were removed and the remaining studies were marked relevant or irrelevant by applying the inclusion criteria (Table 3). This process was supported by ASReview using its default settings (Naïve Bayes classifier, with TF-IDF feature extraction and certainty-based sampling), as these defaults consistently yield good results across various datasets (van de Schoot et al. 2021).

**Table 3:** Inclusion and exclusion criteria used in our review that aims to position studies regarding the combination of Digital Maturity and Systemic Depth in wildlife management and conservation

Inclusion criteria	Exclusion criteria
The study **must mention the use of a specific digital system**, not technology or digital systems in a general sense, in its title or abstract. [IN1] The study **must mention an issue in wildlife management and conservation practices** in its title or abstract, and not be just a technical challenge or merely an observation, for example, of animal behaviour or an environment. [IN2]	The study **is not written in English**. [EX1] The study **is not an article, conference paper or book chapter**, but for example a review, secondary research, or meta-analysis. [EX2] The study **does not pursue a practical objective addressing an issue in wildlife management and conservation**, but for instance mentions it as a potential future use case. [EX3] The study **did not apply a digital system in the field**, but for instance mentions them only, applies the system in a lab setting, or uses the results of another study that did apply a digital system. [EX4]

Active learning systems, such as ASReview, assist reviewers by continuously reorganizing the list of studies to prioritize the most relevant ones. This can lead to a situation where the reviewer has identified nearly all relevant studies, but the list is not yet exhausted, making further evaluation unnecessary. To determine when to stop, we used a predefined “stopping rule” based on the estimated the total number of relevant studies and halted evaluation when 95% of that estimate was reached (Cormack and Grossman 2016). To estimate the number of relevant studies *R*, we performed a random initiation using Eq. (1) in which *N* is the total number of studies, *r* is the number of studies marked relevant during this initiation, and* i* is the number of studies marked irrelevant until *r* is reached (van Haastrecht et al. 2021).

1 $$R \approx N \times \frac{r}{r+i}$$

Formula to estimate the number of relevant studies (R) within the total number of studies (N) using a random initiation with relevant studies (r) and irrelevant studies (i) (van Haastrecht et al. 2021)*.*

A universal method for setting *r* is lacking. The value of 10 is suggested for simplicity, with adjustments possible based on the expected prevalence of relevant studies (Cormack and Grossman 2016). The consensus is that *r* should be proportional to the total set size (*N*), avoiding values that are too small (which risk missing relevant studies) or too large (which diminish the efficiency of active learning). Therefore, *r* should be relative to *N* and account for the expected prevalence of relevant studies. Given no specific expectations for prevalence, we set *r* to 55, or 1% of *N*. This is significantly higher than the 10 suggested by Cormack and Grossman, enhancing the rigor of the learning system’s random initiation, albeit increasing the time required.

**Fig. 2:**
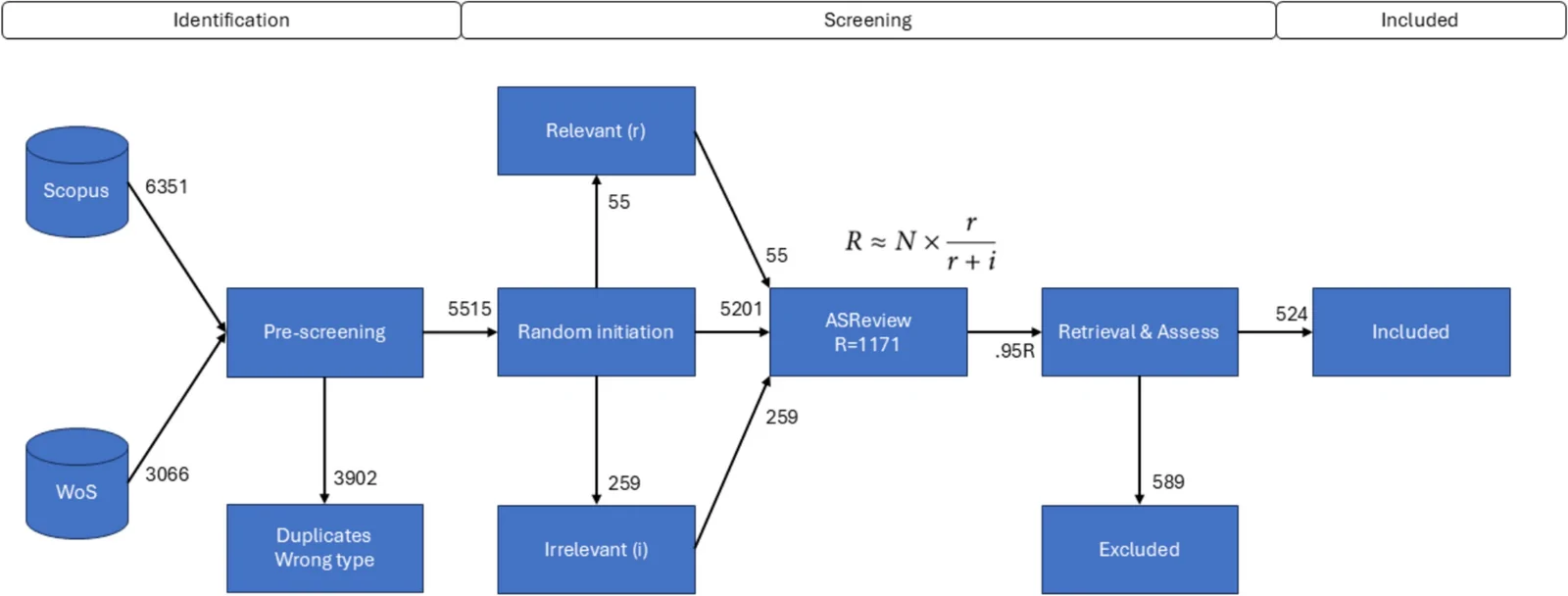
Our flow diagram based on the PRISMA 2020 flow diagram (Page et al. 2021), adapted to include ASReview

In this process, shown in Fig. 2, *N* was 5515, *r* was set to 55, and *i* turned out to be 259 after the random initiation, resulting in an estimated *R* of 1171. Evaluation stopped when 95% of 1171, namely 1112 relevant studies, were found. The next step of the screening process involved retrieving the 1112 relevant studies and manually applying the exclusion criteria. This resulted in excluding 71 studies because the articles could no longer be accessed, and excluding 517 studies based on the exclusion criteria: EX1 (34), EX2 (55), EX3 (145), and EX4 (283). In total, 589 studies were excluded and 524 studies were included in this review.

The retrieval of the 1112 studies, along with reading the articles, applying exclusion criteria, and collecting data from included studies (see coding scheme below), was conducted by a team of 11 reviewers. The first author (BM) processed 152 articles, while the other reviewers (IA, JB, MF, TH, AN, MT, HV, EW, WY, SZ) each processed 96 articles. To standardize the reviewing process, the team held a kick-off meeting to define key concepts and methods, resulting in an agreed-upon Reviewing Guideline (Online Resource 1). Throughout the screening process, four additional calibration meetings addressed issues or uncertainties raised by the reviewers. Given the diverse academic backgrounds of the reviewers, these calibration sessions primarily aimed to clarify definitions and update the reviewing guideline with examples from the processed articles, ensuring the use of consistent terminology. Additionally, to assess evaluation consistency among reviewers, a random sample of 10% of the 1112 studies (111 articles) was selected for double review by different reviewers. The calculated field *Transformative Potential*, ranging from 0 to 16, was used as an indicator of reviewer agreement. The mean absolute difference in scores between the double-reviewed articles was calculated to be 1.11, representing 6.9% of the total score range. This relatively small difference indicates a high level of inter-rater reliability, suggesting that the evaluations among reviewers are closely aligned.

After all studies were processed, BM merged all data using Python (v3.12.7) and Pandas (v2.2.2) and exported them for storage in a FAIR compliant data repository. No missing data was imputed, nor was any data changed in any way that would change its meaning; however, some fields were unified in format (i.e., Latitude and Longitude) or in the use of casing or list separators.

### Coding scheme

We defined 23 data fields (Table 4), of which 14 served to review the study characteristics and 9 to assess the transformative potential of the reviewed studies using our DSTF. A number of study characteristics are captured by grouped fields: for example one field for a shortlist of options and another for “others” in free text, or one more specific and another more general, like *Wildlife Species* and *Taxonomic Classes*. We grouped the fields *Continent*, *Country*, *Latitude*, and *Longitude*, all related to location. While values for the latitude and longitude of the study location could suffice to infer the country and continent, including all four fields allows for varying levels of detail. Some studies provided data on the continent and/or country without latitude and longitude, or vice versa, so this approach ensures we capture location information in all cases.

**Table 4:** The 23 data fields used during the process of data extraction, 14 fields are used for the study characteristics, of which some grouped, 4 fields are used for Digital Maturity and 5 fields are used for Systemic Depth

	Data fields	Group
Study characteristics (14 fields)	Used digital systems, Other digital systems, Intended use, Other intended uses, Wildlife species, Taxonomic classes, Type of end-user, Other end-users, Continent, Country, Latitude, Longitude Mentions co-creation with end-users, Mentions transformational potential	Digital systems Intended use Wildlife End-users Location
Digital Maturity (4 fields)	Visualizations, Statistical modelling, Machine learning, Data storing/sharing	
Systemic Depth (5 fields)	Parameters, Feedback, Design, Intent, Explains leverage point interactions	

**Fig. 3:**
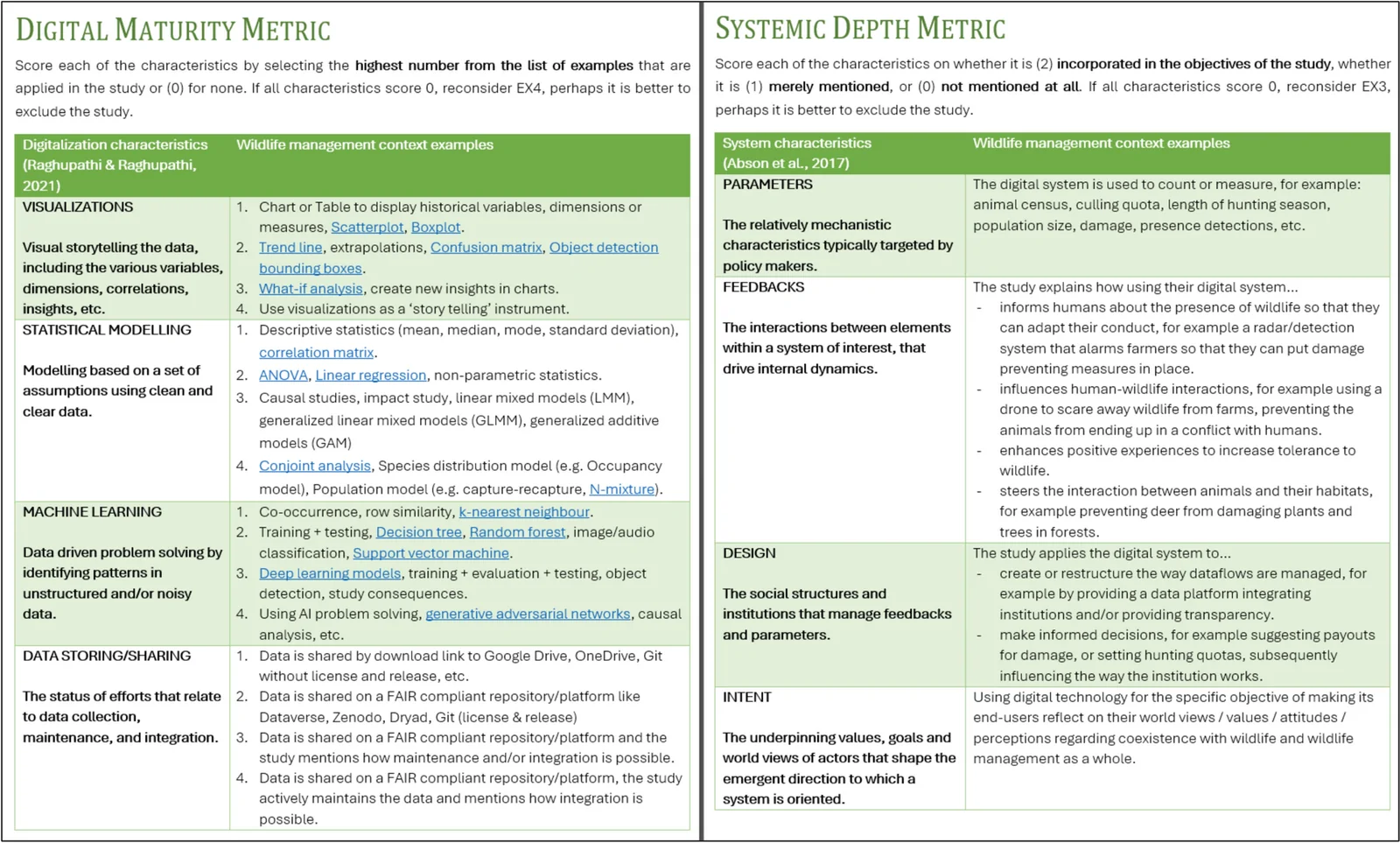
The wildlife management and conservation examples we created for the digitalization characteristics and the system characteristics, used by eleven reviewers during coding

Digital Maturity is defined by four digitalization characteristics that are scales. These scales allow for scoring based on the extent to which each characteristic is incorporated in the study, and examples for each of the levels 1–4 are given by Raghupathi and Raghupathi (2021). However, because the three pillars from which we adopted the characteristics were originally designed to identify business analytics using typical business data such as sales numbers and trend lines, we extended some of those examples to match the context of wildlife management and conservation, in which typically images and sounds are more prevalent types of data. These examples (Fig. 3) were then added to the Reviewing Guideline and shared with all reviewers. All four digitalization characteristics were marked with the number of the example (1, 2, 3, or 4) that best reflects the study’s conduct. If the study did not utilize any examples, it yields a score of 0 for that characteristic. If multiple examples are used, the characteristic obtains the highest score among them. The value for Digital Maturity, with a range 0–16, is then calculated using Eq. (2).

2 $$Digital\;Maturity = Visualization + Statistical\;Modelling + Machine\;Learning + Data\;Storing/Sharing$$

Calculation of Digital Maturity based on the digitalization characteristics.

Systemic Depth is defined by four system characteristics that are evaluated based on its incorporation in the study’s objectives, it being mentioned, or its absence. To derive explicit examples for wildlife management and conservation, we have drawn upon the work of Hartel et al. (2019), who applied the four system characteristics to the context of human-large carnivore coexistence and provide clear context examples. We generated analogous examples for wildlife management and conservation (Fig. 3), which were then added to the Reviewing Guideline and shared with all reviewers.

The system characteristics defining Systemic Depth have an inherent order: *Parameters* are easier to address and possess less transformational power than *Feedbacks*, which in turn are considered of less transformative potential than *Design*, and *Intent*. Incorporating a system characteristic associated with deeper leverage points results in progressively higher scores (2 for *Parameters*, 3 for *Feedbacks*, 4 for *Design*, and 5 for *Intent*). Merely mentioning a characteristic always yields a score of 1, irrespective of the system characteristic addressed. Not mentioning a system characteristic results in a score of 0.

Additionally, we included a field to indicate whether a study explains the interactions between addressed leverage points, as it is important to adopt a systemic view of sustainability issues rather than treating them as discrete elements (Abson et al. 2017). Thus, studies that adopt a “systems thinking” approach to leverage points have greater transformational potential, which must be reflected in their positioning on the DSTF. A score of 2 is assigned to studies that explain these interactions, while a score of 0 is given otherwise. The value for Systemic Depth, with range 0–16, is then calculated using Eq. (3).

3 $$\begin{aligned} Systemic\;Depth & = Parameters \\ & \quad + \left( {3\;if\;Feedbacks = 2\;else\;Feedbacks} \right) \\ & \quad + \left( {4\;if\;Design = 2\;else\;Design} \right) \\ & \quad + \left( {5\;if\;Intent = 2\;else\;Intent} \right) \\ & \quad + 2*Explains\;LP\;Interactions \\ \end{aligned}$$

Calculation of Systemic Depth based on the system characteristics and whether the study explains the leverage point interactions.

### Data analysis

In our systematic literature review, we focused on six key characteristics that emerged from the analysis of the 14 study characteristics. These characteristics encompass the countries of the studies, the biomes of the study areas, the digital systems employed, the taxonomic classes and species of the animals studied, the intended uses of the digital systems, and the types of end-users and their degree of involvement in co-creating the digital system.

To understand the environmental contexts of the studies, we extracted location information and the name of the country in which study was carried out. We then utilized the Terrestrial Ecoregions of the World (Olson et al. 2001) to categorize each study area by its biome. For locations that fell outside terrestrial ecoregions, we classified them under the Marine biome. This classification provided a comprehensive overview of the ecological settings in which the studies were conducted. We also compiled a list of digital systems mentioned in the abstracts of the reviewed studies. This process allowed us to create a shortlist of similar digital systems and quantify their usage across the studies, recognizing that individual studies may incorporate multiple systems. Furthermore, we collected both species names and taxonomic classifications of the studied animals. To streamline our analysis, we grouped *Osteichthyes* and *Chondrichthyes* under the term “Pisces”, reflecting the similarities in the digital systems used for their management and conservation. Lastly, we developed shortlists for the Intended Uses and Types of End-users, and added the options ‘Other Intended Uses’ and ‘Other End-users’. The minimal use of these “other” categories indicates that our shortlists were comprehensive, effectively capturing the relevant information regarding the applications and stakeholders involved in the digital systems.

To assess the transformative potential of the notable literature contributions, we calculated the Systemic Depth, the Digital Maturity and the Transformative Potential for every study, the latter being the geometric mean of the other two. We positioned the Transformative Potential of every study on the DSTF to visualize the results. Additionally, we investigated the prevalence of values for each of the scoring metrics that contributed to the aforementioned scores using histograms, as this helps understand the underlying structure of the data, the central tendency, variability, and shape of the distribution.

To gain a deeper understanding of the factors contributing to Transformative Potential, we complemented our quantitative analysis with a thematic analysis of the top-scoring studies. We selected studies that exceeded the 99th percentile of Transformative Potential, effectively identifying the top 1% of studies with the highest scores. These articles were re-read by the first author (BM), who followed a practical guide for qualitative research (Ayton et al. 2023) to apply inductive coding, extracting paragraphs that were deemed supportive to the concept of Transformative Potential by mentioning activities to ‘transform’, ‘change’, ‘improve’, etc. We then summarized and coded those paragraphs and conducted thematic analysis (Braun et al. 2019) by grouping the codes into themes of meaning. We then associated these themes to the data fields to identify which of the field values are possible predictors for structural factors that contribute to higher transformative potential. We fitted robust linear regression models, with the transformative potential score as the dependent variable and the selected data field values as independent predictors (Online Resource 2). Robust regression models were chosen to mitigate the influence of outliers.

## Results

### The use of digital systems in wildlife management and conservation

**Fig. 4:**
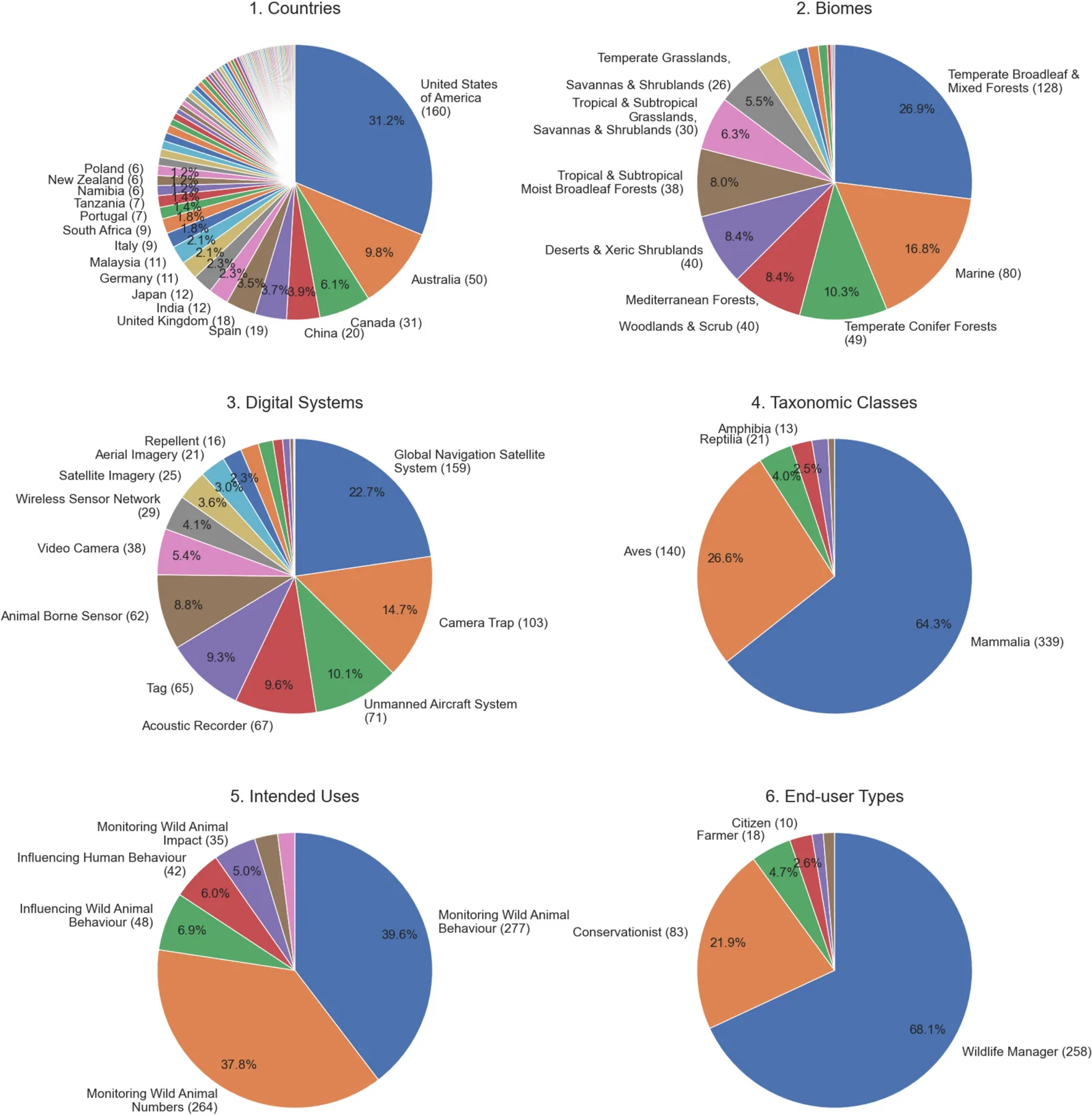
Overview of the study characteristics identified in the reviewed studies, presented as pie charts arranged from top left to bottom right. The six characteristics include: (1) countries of study location, (2) location associated biomes, (3) types of digital systems utilized, (4) taxonomic classes of the studied wild animals, (5) intended application uses, and (6) types of end-users

To provide an overview of how digital systems are used in the wildlife management and conservation we focussed our analysis on the previously mentioned six key characteristics. As shown in Fig. 4, the United States of America was the country with by far the most reviewed studies (31.2%), followed by Australia (9.8% of studies) and Canada (6.1%). In terms of biomes, most studies were located within the Temperate Broadleaf & Mixed Forests (26.9%), followed by Marine (16.8%) and Temperate Conifer Forests (10.3%). Of all the mentioned digital systems, Global Navigation Satellite Systems (GNSS) were applied most frequently (22.7%), followed by Camera Traps (14.7%), Unmanned Aircraft Systems (10.1%) and Acoustic Recorders (9.6%). Mammals were by far the most extensively studied taxonomic class (64.3%), followed by birds (26.6%), and the remaining taxonomic classes were represented in the literature to a much lesser extent. Monitoring Wild Animal Behaviour (39.6%) and Monitoring Wild Animal Numbers (37.8%) together cover more than three quarters of the provided use cases, whereas all the other intended uses together defined the remaining part. A large majority of the mentioned end-user types were Wildlife Managers (68.1%) and Conservationists (21.9%), followed by Farmers (4.7%). When looking merely at the 27 studies that explicitly mention that their digital system was developed in co-creation with the intended end-user, we observe again predominantly Wildlife Managers being mentioned, but now followed by Citizens (18.5%), Conservationists (14.8%), and Farmers (11.1%). For each of the six study characteristics there is also a bar chart available in the Analysis notebook (Online Resource 2), showing all values for every characteristic.

**Fig. 5:**
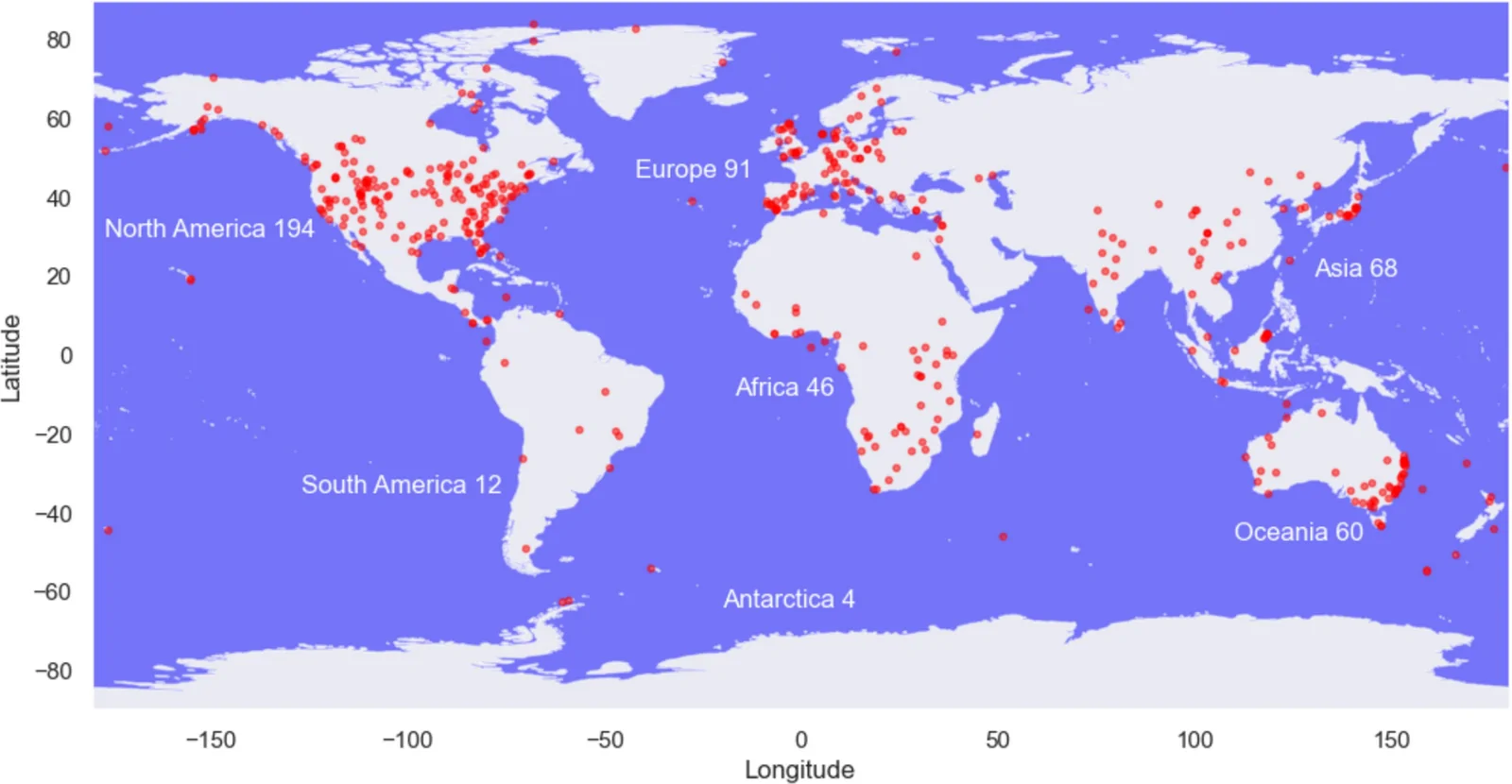
Map of the world with 475 studies areas showing a high density of studies in North America, Europe, Australia and Japan

Out of the 524 studies reviewed, 475 provided data on their study areas, with the majority located in North America, Europe, Australia, and Japan. In both Africa and Asia, study areas seem to be concentrated in parts of the continents, southern and eastern Africa, China, and India specifically. Additionally, only a few studies were located in South America (Fig. 5).

**Fig. 6:**
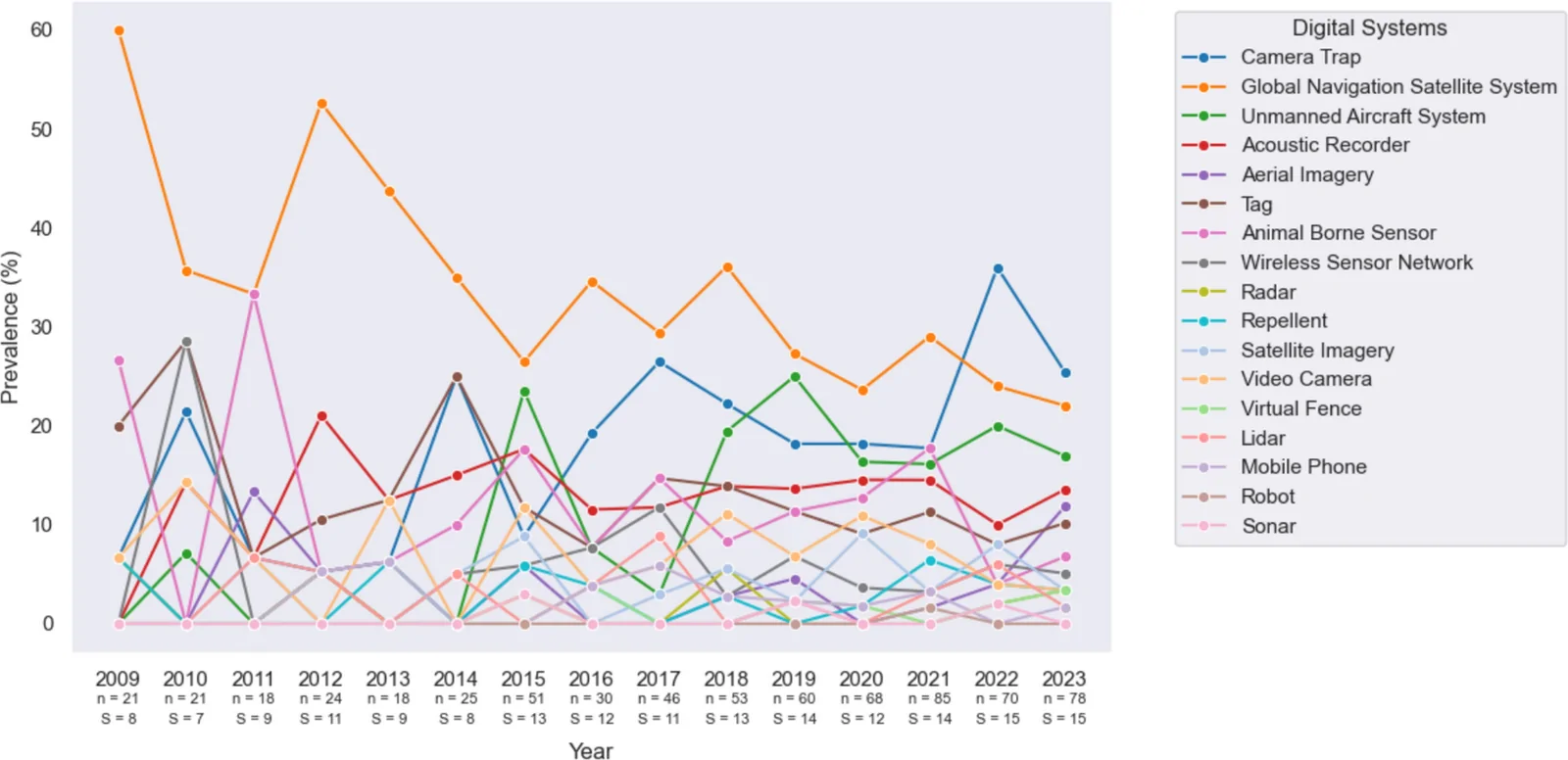
Trends in the utilization of 17 digital systems over the past 15 years, highlighting the significant increase in diversity and application. The data illustrates a near tripling of utilization (n), from 21 in 2009 to 78 by 2023 and a near doubling of distinct systems (S) from 8 in 2009 to 15 by 2023. The legend is ordered by the 2023 data

Our analysis revealed a substantial increase in both the utilization and diversity of digital systems employed in wildlife management and conservation in the last 15 years. Specifically, there were 21 references to digital systems, comprising 8 distinct types, in 2009, compared to 78 references, including 15 distinct types, by 2023 (Fig. 6). Initially, Global Navigation Satellite Systems were the most frequently used digital technology; however, their usage has decreased in recent years. In the early 2010s, the use of Camera Traps exhibited variability, with fluctuations in their application across different years. Beginning in 2015, the adoption of Camera Traps stabilized, and by 2022, their usage surpassed that of Global Navigation Satellite Systems, establishing them as the most widely utilized digital system in the reviewed literature.

### Transformative potential of reviewed literature

**Fig. 7:**
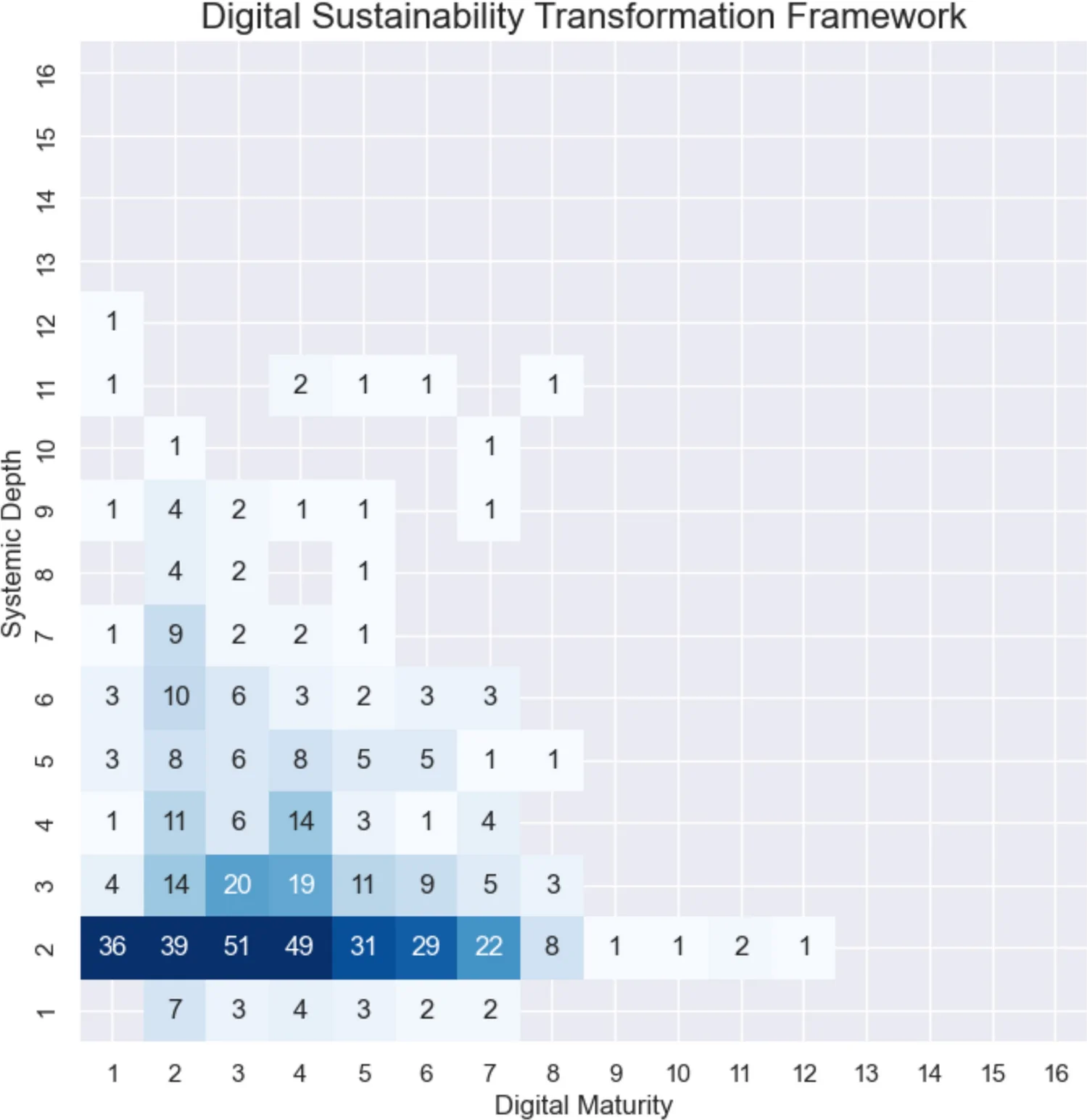
Digital Sustainability Transformation Framework for the total of 524 studies exhibiting a right-skewed pattern for both Systemic Depth Score (γ = 1.818) as well as Digital Maturity Score (γ = 0.703). The values within the cells indicate the number of studies with this score combination

We assessed 524 studies using the Digital Sustainability Transformation Framework (DSTF) (Fig. 7). At a scale of 0–16, the mean Systemic Depth was 3.23 (std = 2.01) and the mean Digital Maturity was 3.80 (std = 1.96). The distribution of Systemic Depth exhibits a pronounced right-skewed pattern, with a skewness coefficient of 1.818, indicating that the majority of studies are concentrated below the mean (71.8%), while a smaller proportion of studies score substantially higher. In contrast, the distribution of Digital Maturity is also right-skewed, but to a lesser extent, with a skewness coefficient of 0.703. This indicates a more balanced distribution, with a smaller proportion of studies scoring below the mean (48.9%). Only 15.5% of the studies mention the transformative potential of their study in relation to the study objectives. Additionally, except for just one study that scored in both the top half of the Digital Maturity scale as well as in the top half of the Systemic Depth Scale, all studies that score in the top half of the Digital Maturity scale, score in the lower half of the Systemic Depth scale, and vice versa.

Looking at Digital Maturity, the findings presented in Fig. 7 indicate that almost all studies utilized *Visualizations*, with the majority employing descriptive visualizations (65.8%). Regarding *Statistical Modelling*, a somewhat linear relationship is evident, suggesting that as the complexity of the modelling techniques increases, their prevalence decreases, with 19.9% of the studies scoring 3 or 4. A distinct pattern emerges for *Machine Learning*, where the simplest machine learning solutions are no longer frequently utilized, and the more complex solutions appear to receive greater interest, with 18.7% of the studies employing more advanced machine learning or deep learning solutions. However, most of the reviewed studies did not implement any machine learning solutions at all (78.8%). Although the digitalization characteristic *Data Storing & Sharing* remains unaddressed by 82.4% of the studies, when it is addressed, studies tend to invest considerable effort, achieving scores of 2 or higher more often than a score of 1.

Looking at Systemic Depth, the system characteristic *Parameters* was included in the study objectives of 93.5% of all reviewed articles. While the *Feedbacks* characteristics within a system were frequently mentioned (26.7%), they were seldom addressed in the study objectives (13.4%). A similar pattern is observed for the characteristics regarding a system’s *Design* (15.1% and 7.4%, respectively). A system’s *Intent* was both rarely mentioned (7.4%) and incorporated (0.6%). Additionally, only 4.2% of the reviewed studies included an explanation for the interactions between the different levels of systemic depth (Fig. 7).

### Traits of studies with high transformative potential

**Fig. 8:**
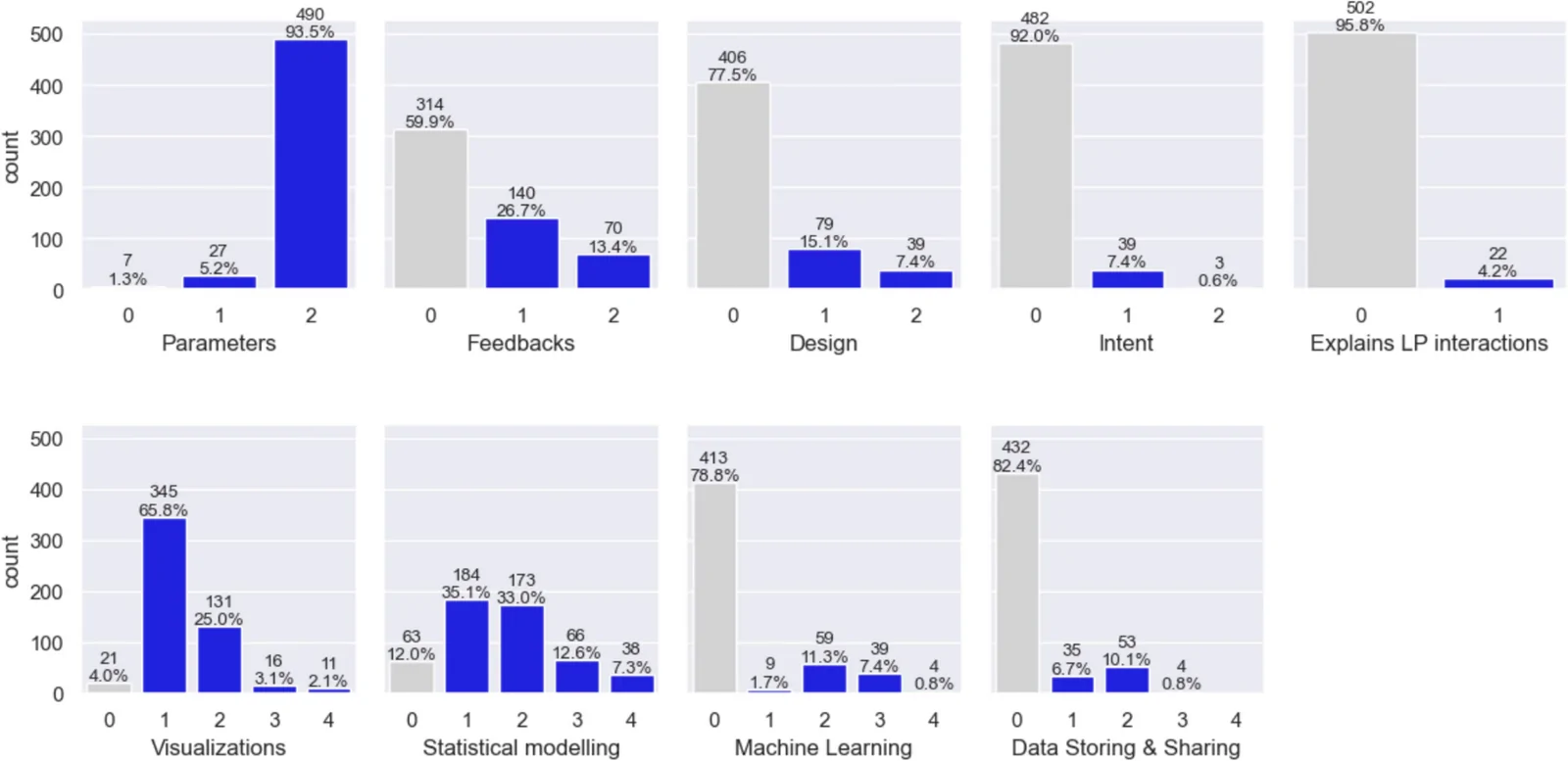
Histograms for the 524 studies, illustrating the prevalence of the values associated with the fields utilized in the calculation of the Systemic Depth, the Digital Maturity and the Transformative Potential, showing the central tendency, variability, and shape of the distribution of the underlying Data

We identified three key themes among the 1% studies with the highest Transformative Potential from the Thematic Analysis (Online Resource 3): interdisciplinary/collaborative approaches, adaptable and responsive strategies, and the significant impact of human activity on wildlife (Fig. 8).

One of the primary themes is the importance of interdisciplinary and collaborative approaches in wildlife management and conservation efforts. This is evident in the emphasis that these studies put on working in interdisciplinary teams, engaging with citizen scientists, and creating community involvement. By bringing together diverse stakeholders and expertise, these studies aim for more effective and sustainable wildlife management and conservation outcomes, by leveraging a broader range of skills and knowledge. Based on this theme we examined data fields *Type of end-user* and *Mentions co-creation with end-users* as predictors for *Transformative Potential*. Robust linear regressions (Online Resource 2) show that the type of end-user mentioned in a study is positively associated with its score for Transformative Potential (scale 0–16). When the end-user is described as a professional (conservationist, ecologist, wildlife manager) the score rises by 0.69 points (95% CI 0.50–0.89, *p* < 0.001) compared with studies that do not mention any end-user. A separate model for studies that name community actors (citizens, farmers, foresters) yields an even larger increase of 1.03 points (95% CI 0.66–1.40, *p*  < 0.001) relative to the “none” reference group. In addition, the binary indicator *Mentions co-creation with end-users* is associated with a 0.57 points higher score (95% CI 0.14–1.00 *p* = 0.009). These results indicate that explicitly identifying the target end-user, particularly community stakeholders, and reporting co-creation are structural factors linked to higher transformative potential.

**Actionable insight 1**: We suggest conducting applied research in an interdisciplinary way by combining different scientific disciplines and involving practitioners and citizens to collaborate in designing and conducting wildlife management and conservation studies.

Another significant theme that emerges from these studies is the need for adaptability and responsiveness in wildlife management and conservation. This is reflected in the emphasis on adapting to rapid changes, often using a combination of different digital systems to gather richer information, as well as integrating data from various sources to optimize models and information availability. Moreover, the studies indicate that the use of modern technologies, such as machine learning, also plays a crucial role in increasing the capacity of data gathering efforts, effective monitoring, and management decision making. Consequently, the importance of data quality and sharing is highlighted, with many studies emphasizing the need for high-quality, expert-curated data, and a willingness to share their data in a FAIR compliant way. Associating this theme to the number of *Digital systems used*, we found that using more digital systems yields a modest increase in the score for Transformative Potential of 0.19 points per additional system (95% CI 0.04–0.35, *p* = 0.016). This theme is also associated with the data fields *Machine learning* and *Data Storing & Sharing* and thus inherently implies higher Transformative Potential, as these data fields are components of the score for Digital Maturity.

**Actionable insight 2**: We suggest integrating multiple, complementary data sources to create a richer, higher-quality view on wildlife dynamics, processed by cost-effective machine learning pipelines that deliver consistent analyses and can be scaled up as more data become available.

Lastly, these top-scoring studies also highlight the significant impact of human activity on wildlife, with themes such as human noise and pollution, roads, and other anthropogenic factors affecting animal welfare. In response to these challenges, the studies emphasize the need for technology to monitor and understand the impact of human activity on wildlife, as well as the importance of involving and educating the community in wildlife conservation efforts. We associated this theme to the value ‘Influencing Human Behaviour’ of the field *Intended uses* and found that studies explicitly stating an intention to influence human behaviour obtain a transformative potential score that is on average 1.11 points higher (95% CI 0.78–1.44, *p* < 0.001) than studies that do not mention this intention. This suggests that recognising human behaviour as a structural factor and embedding it in the study design is linked to a higher transformative potential.

**Actionable insight 3**: We suggest mitigating the adverse effects of human activities on wildlife by levering community engagement, citizen science, and education in order to rethink human-wildlife relations.

## Discussion

In this study, we conducted a systematic literature review examining the application of digital systems in wildlife management and conservation, using our Digital Sustainability Transformation Framework (DSTF) to assess the transformative potential of the 524 reviewed studies. Although some studies reach higher scores on Digital Maturity or Systemic Depth, combined efforts to increase transformative potential remain rare. Moreover, only a very small number of studies explicitly mentioned ‘transformation’ or ‘transformative potential’ in relation to their study objectives.

### The use of digital systems in wildlife management and conservation

Our findings indicate that digital systems in wildlife management and conservation are primarily employed to study larger mammals, with a secondary focus on birds. However, our reliance on the term “wildlife” in the search strategy may have led to the underrepresentation of studies across various taxa, including ornithological and herpetological studies, particularly in cases where researchers did not explicitly use “wildlife” as a descriptor. This terminological difference may have skewed the apparent taxonomic focus in our findings. While our review provides a comprehensive overview of the current state of digital systems in wildlife management and conservation, it also highlights some gaps in our understanding that may be opportunities for further investigation, for example the previously mentioned taxa, or particular countries or biomes that have not been captured well by our review. Additionally, we are unable to offer guidance on the effectiveness of specific digital technologies or on how analytical approaches using these technologies influence systemic transformation because we did not assess the extent to which the reviewed studies achieved transformative outcomes. We believe this presents a promising avenue for future research on the use of digital technologies in wildlife management and conservation studies.

Digital systems that have garnered the most interest from the wildlife management and conservation community in recent years (camera traps, acoustic recorders, and unmanned aerial systems), are designed to collect visual and/or audio data. Therefore, it is reasonable to infer that research efforts are concentrated on species that are visually detectable against a large variety of different backgrounds (larger mammals) or well-known for producing distinctive sounds (birds). As a consequence, these digital systems have largely been used for monitoring purposes (detecting where animals occur and how many there are), which might explain the low Systemic Depth scores. The use of these digital systems may have been constrained by the substantial volumes of data they generate, which require extensive filtering and labelling (Tuia et al. 2022). However, in more recent years, these tasks have become easier to handle thanks to advancements in machine learning and deep learning technologies, thereby creating new opportunities for wildlife monitoring and conservation through the use of these digital systems (Gewin 2025; Pollock et al. 2025). These new developments will likely also increase the potential for digital systems to be used in a wider array of uses, such as influencing wildlife behaviour (Widén et al. 2022) and real-time feedback loops between wildlife and humans (Astaras et al. 2020).

Recent years have seen a surge of national-scale digital infrastructure initiatives responding to the alarming global decline in biodiversity (IPBES 2019). These programmes aim to build comprehensive, end-to-end biodiversity data platforms that go beyond plain data aggregation (Güntsch et al. 2025). They provide advanced services such as automated species identification, interactive dashboards presenting region-specific trends, and data-informed recommendations to support local biodiversity improvements. Importantly, these platforms are designed not only for nature conservation professionals but also for researchers, citizen scientists, and commercial stakeholders. A prominent example is the ARISE project in the Netherlands (van Ommen Kloeke et al. 2025), a largescale initiative that delivers an integrated national biodiversity infrastructure. Notably, ARISE also hosts an open repository where scientists can upload and benchmark improved AI-based solutions across a wide range of floral and faunal taxonomic groups.

### Transformative potential of reviewed literature

In contrast to Systemic Depth, where most of the 524 reviewed studied scored between 2 and 3, we found the Digital Maturity scores to span a larger area, ranging from 2 to 8. This discrepancy implies that although different digital solutions are used, they are rarely aimed at leverage points beyond the system characteristic *Parameters*, a finding that could be linked to a phenomenon known as “techno-fixing” (Sætra and Selinger 2023). Techno-fixing can be described as turning a social problem into an engineering challenge followed by providing a solution to this engineering challenge claiming it alleviates the social problem. Alvin Weinberg first defined and advocated for techno-fixing in 1966 (Weinberg 1966), but since then received increasingly more criticism as techno-fixes often bypass the social basis of the problem and provide a solution that addresses only the symptoms.

Using techno-fixes is not restricted to wildlife management and conservation but is prevalent also in other fields, for example in healthcare (Jongsma and Sand 2017), food and energy systems (Dorninger et al. 2020), indigenous food systems (Zimmermann et al. 2023), marine and coastal pollution (Riechers et al. 2021) and justice (Wells 2008). In every field, techno-fixing presents appealing but flawed solutions to real-world problems. These solutions demonstrate that digital devices are excellent at gathering data, but the availability of these data can lead to management strategies that oversimplify complex issues, neglect ethical concerns regarding abuse or access limitations, and create inequity.

In wildlife management and conservation, focus on the system characteristic *Parameters* may increase the likelihood of overlooking other aspects of these digital systems that address deeper leverage points. Moreover, it prevents a reflection on the impact of technology on human-wildlife interactions. Studies within the social sciences have shown the use of digital technologies could help people move closer to nature through connection and stewardship but could also lead to increased disturbance or surveillance of both wildlife and humans (Verma et al. 2015; Turnbull et al. 2023; von Essen et al. 2023). How technology is used and the impact it can create in wildlife management depends on the visions or imaginaries behind the deployment of these technologies (Gabrys et al. 2025). The environmental data collected through digital systems in wildlife management are often seen as objective information that informs policy. However, this data is not neutral and can be seen as a ‘site of control’, where choices determine who can be involved, what gets produced and which decisions can be made (Nost and Goldstein 2022). While researchers and managers are often aware of these tensions that come with using digital systems (Arts et al. 2015; Verma et al. 2015), without the consideration of deeper leverage points, conservation technologies will not be able to address issues of extraction and control, and transform beyond ‘business as usual’.

It is inevitable that new digital technologies of the techno-fix type are applied to simpler issues first, as their novelty often brings inherent risks or teething problems, and end-users may need to acquire new skills before they can effectively leverage the technology to tackle more complicated challenges. Additionally, it is understandable that it is tempting to purchase ready-made digital technologies instead of codeveloping and finetuning technology to specific end-user needs. However, as digital technologies mature, their application to more complex social contexts requires a critical reassessment of their objectives, because the goals that were relevant in simpler contexts may not be directly applicable to more complex social issues, potentially leading to mismatches between the technology’s intended purpose and the actual problem it needs to address. Utilizing our DSFT identifies these limitations by providing a structured tool to characterize existing research and system interventions across various levels of Systemic Depth and Digital Maturity. By recognizing the nested hierarchy inherent in Systemic Depth, our DSFT highlights areas where systemic change is either constrained or facilitated. This understanding is essential for future research and the design of more effective interventions using digital systems, as it ensures that efforts are not overly concentrated on shallow leverage points (Scoones et al. 2020).

Studies with higher Digital Maturity scores integrate multiple data collection and modelling techniques, utilize visualizations effectively, and overcome data sharing hurdles, while those with higher Systemic Depth scores demonstrate an ability to challenge existing wildlife management and conservation regulations and laws. While the combination of these characteristics represents an aspirational ideal, it may prove impractical for many studies, potentially limiting their feasibility, which may explain the observed focus on the system characteristic *Parameters*. Although we advocate for incorporating deeper levels, such as stakeholder engagement and citizen science, this shift will require further research to assess the effectiveness of these approaches and to develop strategies for scaling them up and applying them in diverse contexts.

Our DSTF is designed for broad applicability and can be easily recognized and implemented by professionals across various sustainability domains, including urban climate adaptation, regenerative agriculture, biodiversity conservation, and renewable energy systems. Its components are based on established metrics, enabling early assessments of a project’s transformative potential and highlighting opportunities for improvement. However, our DSTF has some limitations that may lead to misinterpretation of the results, notably that the scales used are not guaranteed to be linear, meaning that scoring higher on a certain metric becomes incrementally harder. This non-linearity complicates cross-study comparisons, as a one-point difference can represent varying magnitudes of change depending on the scoring region. Moreover, studies with the same Systemic Depth or Digital Maturity score may have achieved this score through vastly different means. For example, a score of 5 on Systemic Depth can be obtained by incorporating *Parameters* and *Feedbacks* into a study’s objectives, without addressing *Design* and *Intent* at all, or alternatively, by incorporating *Intent* without addressing *Parameters*, *Feedback*, and *Design* at all, which would likely describe very different studies. However, we argue that due to the hierarchical nature of the system characteristics, the latter scenario is unlikely to occur. Although these limitations exist, we believe it does not detract from our encouragement for other researchers to apply and refine the DSTF in their own studies to evaluate its adaptability across diverse sustainability challenges.

### Traits of studies with high transformative potential

Studies that score higher on our DSTF, which we interpret as having greater transformative potential, tend to employ interdisciplinary teams. This is likely because adopting more mature digital practices necessitates participation of IT specialists, and addressing deeper leverage points requires the involvement of non-academic stakeholders. These factors make project management more demanding and collaboration more complex.

However, several studies (Arts et al. 2015; Fritz et al. 2019; Sauermann et al. 2020), including the latest Transformative Change Assessment by the Intergovernmental Science-Policy Platform on Biodiversity and Ecosystem Services (IPBES), underline that including diverse disciplines and practices such as citizen science, when implemented well, contributes positively to sustainable transformations (O’Brien et al. 2025). Similarly, the role of education and the importance of understanding the impact of human activity and rethinking human relations to nature, have also been linked to transformative potential (West et al. 2020; O’Brien et al. 2025).

Examining the top-scoring studies, we noticed that by emphasizing community engagement, education, and political support, these studies highlight the need for a broader societal commitment, recognizing that wildlife management and conservation is not just a technical challenge, but also a social and political one. The sample size of six of the top-scoring group is too small to support statistical inference, and therefore, we interpret these findings as exploratory and descriptive, rather than conclusive. As shown, digital systems are mostly employed at the Parameters level, where variables about wildlife are measured, with little to no reflection on interactions with humans and systemic change. Furthermore, the implications of these findings are important, as they suggest that wildlife management and conservation must be approached as a multifaceted challenge that requires the integration of ecological, social, and technological perspectives, but has not reached its full potential at present. By adopting a more holistic and collaborative approach to wildlife management and conservation, we can develop more effective strategies that address the complex interactions between human and wildlife systems; this would likely involve the development of new technologies and tools, as well as innovative approaches to community engagement and education. It also requires us to reflect on the way technology mediates our practices and relationship with wildlife. This is not just a question of gathering more data, as the data itself can also become a site of contestation, legitimizing or delegitimizing actors and approaches (Gabrys 2016; Nost and Goldstein 2022). Tracking both animals and humans can for example create a need for control that moves us away from sustainably coexisting (Adams 2019; von Essen et al. 2023). Responsible use would therefore include the recognition of the social impact of digital systems, transparency in use and the consideration of proportionality of impact for all people and animals involved (Sandbrook et al. 2021). It asks for an interdisciplinary approach, integrating ecological, social and local knowledge to understand the barriers that keep the field of wildlife management and conservation from moving to deeper transformative levels. Ultimately, our analysis suggests that the most transformative wildlife management and conservation efforts will be those that are able to balance the needs of both humans and wild animals, and that prioritize the sustainable development of technology-supported, coexistence-based strategies.

## Conclusions

This paper presents a first review to look at the transformative potential of digital systems in wildlife management and conservation, and highlights the critical role digital technology plays in wildlife conservation and management, while also revealing the limitations in achieving transformative outcomes. By assessing the reviewed studies using our Digital Sustainability Transformation Framework (DSTF), we found that while there is a growing interest in leveraging digital solutions, the overall transformative potential of the reviewed studies remains relatively low, as evidenced by the mean scores of Digital Maturity and Systemic Depth. This suggests that many existing digital initiatives may not yet be fully equipped to drive significant, lasting change in wildlife management efforts. To enhance the transformative potential of digital systems in wildlife management, it is essential to co-design these systems in collaboration with all stakeholders and end-users, including, for example, rangers, policymakers, recreationists, farmers living in or around natural areas, and the general public. By engaging diverse groups within interdisciplinary frameworks, we can ensure that their perspectives and needs are effectively integrated, thereby nourishing collaboration and creating more inclusive and impactful approaches to wildlife management.

## Supplementary Information

Below is the link to the electronic supplementary material.

Supplementary file1 (PDF 1085 KB)

Supplementary file2 (HTML 2301 KB)

Supplementary file3 (PDF 432 KB)

## Data Availability

The data that support the findings of this systematic literature review are available at https://doi.org/10.24416/UU01-NF1V8Z.
