# Supplementary material for: Transformative potential of digital systems for promoting human-wildlife coexistence: A systematic literature review
Source: Ambio. 2026 May 21;55(12):2836–55. doi: 10.1007/s13280-026-02399-7 (PMC13731620; doi:10.1007/s13280-026-02399-7)
Supplement: Supplementary file 1 [file 13280_2026_2399_MOESM1_ESM.pdf]

Transformative potential of digital systems for promoting human-wildlife coexistence: A systematic literature review

Ambio

Bas S.H.T. Michielsen, Gerard Schouten, Joris P.G. Cromsigt, Remco C. Veltkamp, Irma Arts, Jitse Bijlmakers, Magali Frauendorf, Tim R. Hofmeester, Amy Newsom, Minh-Xuân A. Truong, Helen Verploegen, Els Weinans, Wenjin Yu, Silja Zimmermann, Ine Dorresteijn

Reviewing guideline

Bas S.H.T. Michielsen
Copernicus Institute of Sustainable Development, Utrecht University, Utrecht, Netherlands
School of ICT, Fontys University of Applied Sciences, Eindhoven, Netherlands
Information and Computing Sciences, Utrecht University, Utrecht, Netherlands
hello@bshtmichielsen.eu

INTRODUCTION

This reviewing guideline supports the coding process for the literature review. The studies that are the result of the search query have been pre-screened on title and abstract. Notice that querying or pre-screening did not exclude studies based on the publishing year, so the literature review does not have a focus timeframe.

The literature review itself is on the context of wildlife management and conservation, wherein practices are changing for reasons regarding sustainability as well as the potential paradigm shift from an anthropocentric viewpoint to co-existing with wild animals. Because of this transformation, wildlife management professionals and researchers are looking at digital tools and devices as enablers for improvement. Digitalization brings to the table increased efficiency in time, costs and effort, but also the fact that digital systems operate on discrete states and have a high precision regarding data collection. Ultimately, this data, when used well in terms of collection, maintenance and integration provides opportunities like automation of processes, scalability, and learning from historical measurements to predict future observations. This literature review thus aims to shed light on how the use of data and digital tools in wildlife management and conservation enables the transformation to more sustainable conduct by scoring studies that exhibit both aspects on a Systemic Depth metric as well as on a Digital Maturity metric, together forming the Transformative Potential of the digital system in the wildlife management and conservation context.

Glossary

Digital system

A combination of software and connected hardware devices, that operates on discrete states, and wherein data is stored and processed in binary digits (Dally & Poulton, 1998) (Bolton, 2015).

Wildlife management and conservation

A process that guides interactions and dynamics between wildlife, its habitats, and humans to achieve predefined impacts (Decker et al., 2012) (Raj & Lal, 2013).

Objectives

Our overarching aim is to evaluate the transformative potential of digital systems as applied in wildlife management and conservation studies by conducting a systemic literature review to:

1. examine the use of digital systems in wildlife management and conservation studies;
2. apply our framework to understand how the reviewed literature compares in terms of transformative potential;
3. describe the traits of studies that the framework has identified as having high transformative potential.

Exclusion criteria

EX1: The study **is not written in English**.

EX2: The study **is not an original research article**, **conference paper** or **book chapter**, but for example a review, secondary research, or meta-analysis.

EX3: The study **does not pursue a practical objective addressing an issue in wildlife management and conservation**, but for instance mentions it as a potential future use case.

EX4: The study **did not apply a digital system in the field**, but for instance mentions them only, applies the system in a lab setting, or uses the results of another study that did apply a digital system.

Systemic Depth

Score each of the characteristics on whether it is (2) **incorporated in the objectives of the study**, whether it is (1) **merely mentioned**, or (0) **not mentioned at all**. If all characteristics score 0, reconsider EX3, perhaps it is better to exclude the study.

System characteristics (Abson et al., 2017)	Wildlife management context examples
**PARAMETERS**<br><br>**The relatively mechanistic characteristics typically targeted by policy makers.**	The digital system is used to count or measure, for example: animal census, culling quota, length of hunting season, population size, damage, presence detections, etc.
**FEEDBACKS**<br><br>**The interactions between elements within a system of interest, that drive internal dynamics.**	The study explains how using their digital system…<br>- informs humans about the presence of wildlife so that they can adapt their conduct, for example a radar/detection system that alarms farmers so that they can put damage preventing measures in place.<br>- influences human-wildlife interactions, for example using a drone to scare away wildlife from farms, preventing the animals from ending up in a conflict with humans.<br>- enhances positive experiences to increase tolerance to wildlife.<br>- steers the interaction between animals and their habitats, for example preventing deer from damaging plants and trees in forests.
**DESIGN**<br><br>**The social structures and institutions that manage feedbacks and parameters.**	The study applies the digital system to…<br>- create or restructure the way dataflows are managed, for example by providing a data platform integrating institutions and/or providing transparency.<br>- make informed decisions, for example suggesting payouts for damage, or setting hunting quotas, subsequently influencing the way the institution works.
**INTENT**<br><br>**The underpinning values, goals and world views of actors that shape the emergent direction to which a system is oriented.**	Using digital technology for the specific objective of making its end-users reflect on their world views / values / attitudes / perceptions regarding coexistence with wildlife and wildlife management as a whole.

Additional clarification

Merely mentioning the words “design” or “intent” does not automatically mean that the paper should be scored on those system characteristics, it is paramount that the used digital system is targeting these characteristics.

A few examples to illustrate the decision boundaries between the system characteristics:

a) A digital system that detects whether animals are ill would address the system characteristic Parameters because it is measuring or counting the number of ill animals, however if it consequently informs a veterinarian who can then perform an intervention, it addresses also the system characteristic Feedback. If the study merely mentions this as a possibility it scores 1, if it included it as an objective of the study and implemented it, it scores 2.

b) A study that measures the wellbeing of wild animals is addressing the system characteristic of Parameters, as it is a measurement; if the study also attempts to influence the wellbeing of those animals is it still addressing the system characteristic Parameters because it is doing measurements to compare the difference; however if the study also states that an improved wellbeing of the animals affects other elements, like human wellbeing, the state of affairs in the habitat, etc it is addressing the system characteristic Feedback as well. If it merely mentions these collateral effects it addresses Feedbacks but scores 1, if these effects are included in the objective of the study and are thus addressed in the implementation of the digital system, it scores 2.

c) Some studies that use educational applications have the goal to let its end-users reflect on their worldviews, values, attitudes and perceptions of coexistence with wildlife and wildlife management. If it only articulates this as a goal but does not present a clear implementation, the study scores 1 on Intent. If the study, for example measures the effect of their educational application on its end-users worldviews, values, attitudes and perceptions of wildlife management and report on that change, it scores 2 on Intent. Note that there may also be educational applications with the objective to inform on or create awareness for wildlife management that are more likely to score on the system characteristics Parameters and/or Feedback. Not every educational application automatically addresses the system characteristic Intent simply because it is educational.

DIGITAL MATURITY

Score each of the characteristics by selecting the **highest number from the list of examples** that are applied in the study or (0) for none. If all characteristics score 0, reconsider EX4, perhaps it is better to exclude the study.

Digitalization characteristics (Raghupathi & Raghupathi, 2021)	Wildlife management context examples
**VISUALIZATIONS**<br><br>**Visual storytelling the data, including the various variables, dimensions, correlations, insights, etc.**	1. Chart or Table to display historical variables, dimensions or measures, Scatterplot, Boxplot.<br>2. Trend line, extrapolations, Confusion matrix, Object detection bounding boxes.<br>3. What-if analysis, create new insights in charts.<br>4. Use visualizations as a 'story telling' instrument.
**STATISTICAL MODELLING**<br><br>**Modelling based on a set of assumptions using clean and clear data.**	1. Descriptive statistics (mean, median, mode, standard deviation), correlation matrix.<br>2. ANOVA, Linear regression, non-parametric statistics.<br>3. Causal studies, impact study, linear mixed models (LMM), generalized linear mixed models (GLMM), generalized additive models (GAM)<br>4. Conjoint analysis, Species distribution model (e.g. Occupancy model), Population model (e.g. capture-recapture, N-mixture).
**MACHINE LEARNING**<br><br>**Data driven problem solving by identifying patterns in unstructured and/or noisy data.**	1. Co-occurrence, row similarity, k-nearest neighbour.<br>2. Training + testing, Decision tree, Random forest, image/audio classification, Support vector machine.<br>3. Deep learning models, training + evaluation + testing, object detection, study consequences.<br>4. Using AI problem solving, generative adversarial networks, causal analysis, etc.
**DATA STORING/SHARING**<br><br>**The status of efforts that relate to data collection, maintenance, and integration.**	1. Data is shared by download link to Google Drive, OneDrive, Git without license and release, etc.<br>2. Data is shared on a FAIR compliant repository/platform like Dataverse, Zenodo, Dryad, Git (license & release)<br>3. Data is shared on a FAIR compliant repository/platform and the study mentions how maintenance and/or integration is possible.<br>4. Data is shared on a FAIR compliant repository/platform, the study actively maintains the data and mentions how integration is possible.

ADDITIONAL CLARIFICATION

Visualizations should be about the data that was collected during the study, it does not matter whether that data was created by the digital system in question, and/or by human observation, however images about something else than the collected data, like photographs, a map of the area, a systematic representation of the digital network, etc. are not to be counted as visualizations.

Using a visualization as a 'story telling' instrument means that a single visualization is used to express a highly complex situation and explains the concepts, the issues, data flows, etc. of the situation as to provide a full story. A possible example is an infographic:

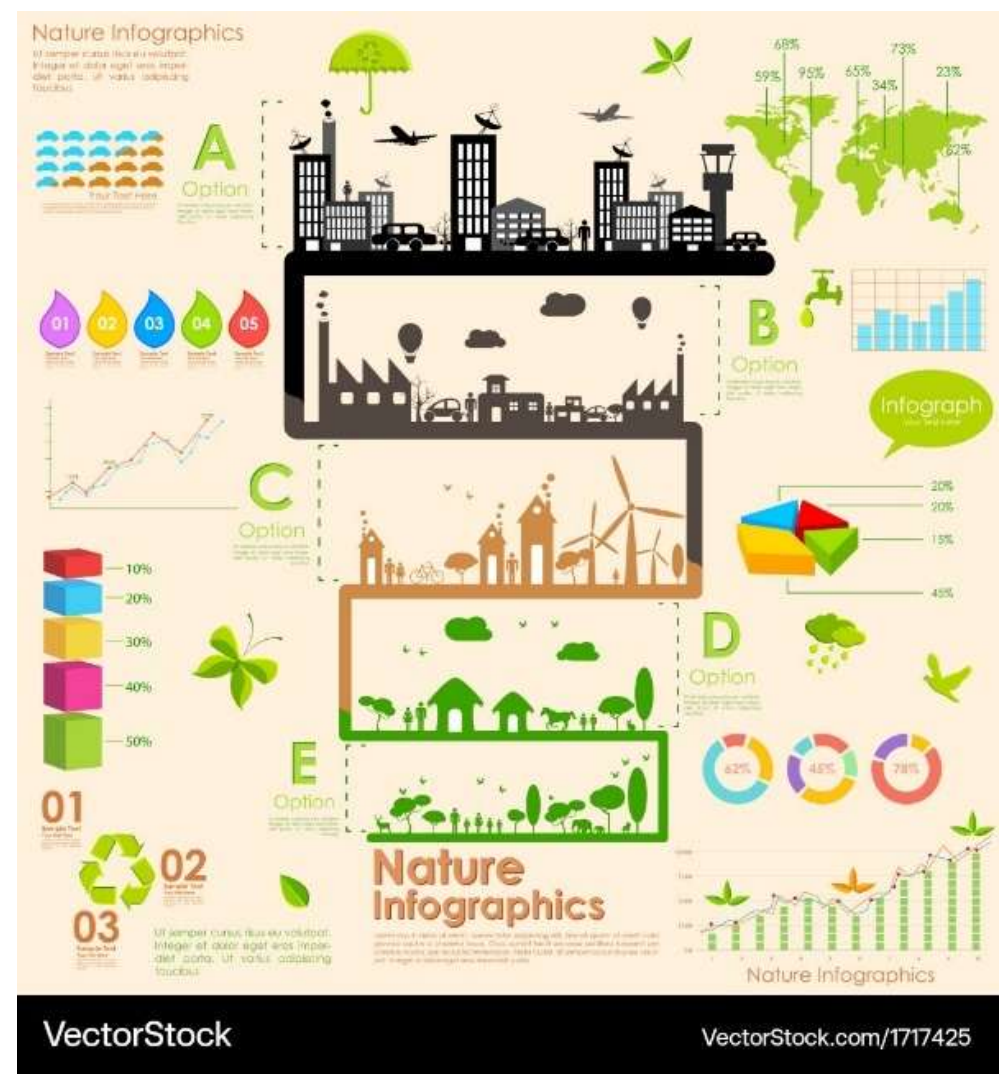

If the data is stored in a data repository not mentioned in the example column, consider whether the dataset was assigned a DOI by the repository. If so, it is likely FAIR compliant and can be coded 2 (or higher), if not, consider whether it is stored in a version control system (like git) and has a license and release. If so, it can be coded 2 (or higher) as it is FAIR compliant by convention. Otherwise it should be coded 1.

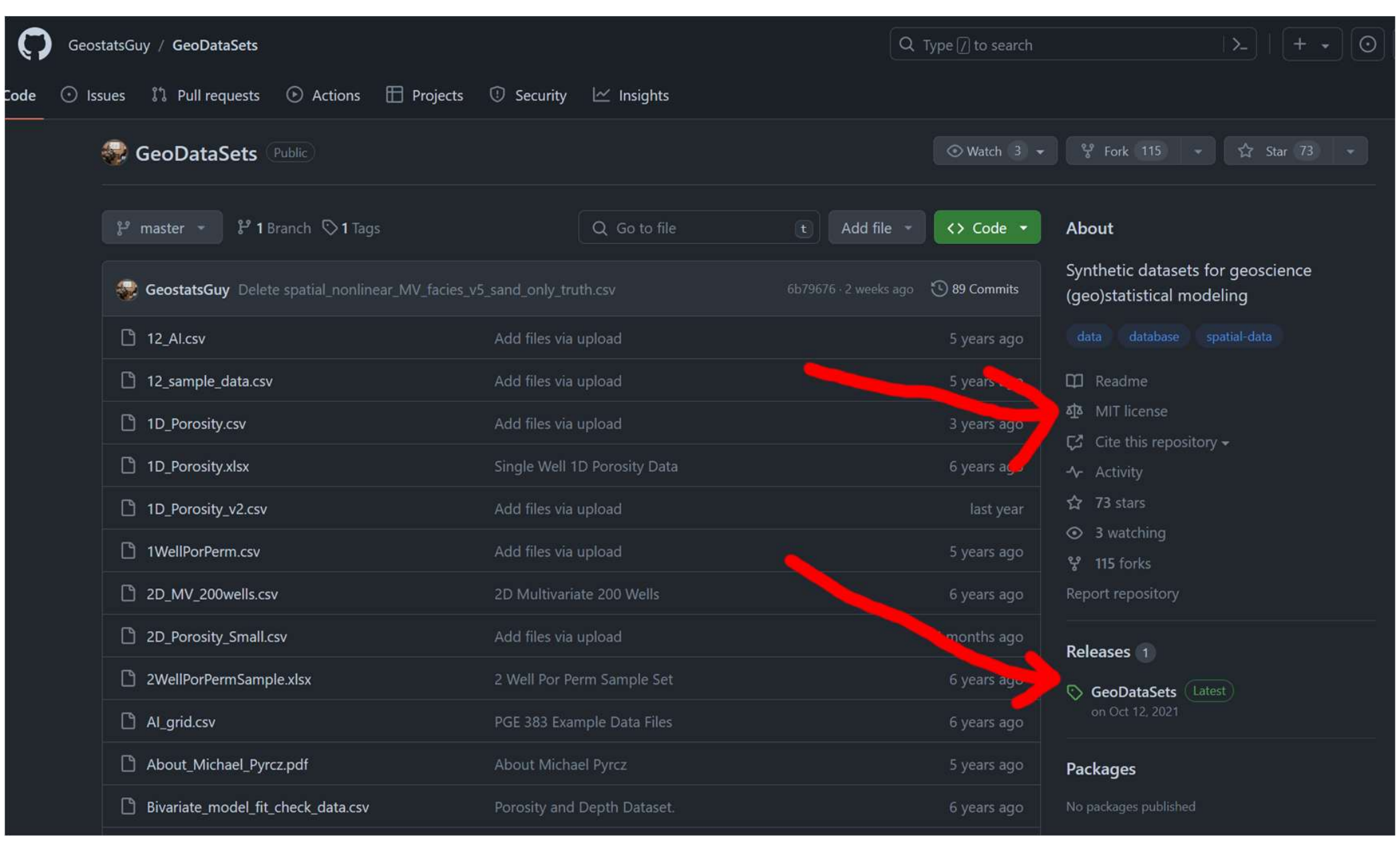

Data fields

Names and data types of the data fields. Curly brackets {} mean 'select exactly one', square brackets [] mean 'select one, many or none' where selecting none of the options means unknown, TEXT is a free text field having "?" for unknown or not applicable, INTEGER is a whole number having 0 for unknown.

Systemic depth

• Parameters	{0, 1, 2}	
• Feedback	{0, 1, 2}	
• Design	{0, 1, 2}	
• Intent	{0, 1, 2}	
• Explains leverage point interactions	{0, 1}	

Digital maturity

• Visualizations	{0, 1, 2, 3, 4}	
• Statistical modelling	{0, 1, 2, 3, 4}	
• Machine learning	{0, 1, 2, 3, 4}	
• Data storing/sharing	{0, 1, 2, 3, 4}	

Study characteristics (as mentioned in the study)

• Intended use	[monitoring wild animal numbers, influencing wild animal numbers, monitoring wild animal behaviour, influencing wild animal behaviour, monitoring wild animal impact, monitoring human behaviour, influencing human behaviour]	
• Other intended uses	TEXT	? = n/a
• Type of end-user	[wildlife manager, conservationist, farmer, forester, citizen, ecologist]	
• Other end-users	TEXT	? = n/a
• Number of end-users in the field	INTEGER	0 = unknown
• Type of human-wildlife interaction	TEXT	? = unknown
• Wildlife species	TEXT (TEXT), TEXT (TEXT) TEXT (TEXT) …	? = unknown
• Taxonomical classes	[mammalia, aves, pisces, reptilia, amphibia, insecta, other]	
• Used digital systems	[uas, camera trap, video camera, gnss, wireless sensor network,	

	acoustic recorder, mobile phone, satellite imagery, aerial imagery, robot, repellent, animal borne sensor, tag, radar, sonar, lidar, virtual fence]	
• Other digital systems	TEXT	? = n/a
• Mentions co-creation with end-users	{0, 1}	
• Mentions transformational potential	{0, 1}	

SPATIOTEMPORAL FACTORS (AS MENTIONED IN THE STUDY)

• Continent	{Africa, Antarctica, Asia, Oceania, Europe, North America, South America}	
• Country	TEXT	? = unknown
• Latitude (decimal notation)	FLOAT	
• Longitude (decimal notation)	FLOAT	
• Size of study area (km^2)	INTEGER	0 = unknown
• Study duration	INTEGER {mins, days, months}	0 = unknown

DOUBT OR ISSUE

• Transformation scoring doubt	{0, 1}
• Digitalization scoring doubt	{0, 1}
• Issue	TEXT

Data field explanations

Further clarification for some of the above-mentioned data items.

Explains leverage point interactions

Mark whether a study explains the way the addressed leverage points interact in a "systems thinking" way. (0) no, (1) yes

Intended use

Note that the short list is based on a categorisation of the intended uses as found during the abstract screening, and it is therefore believed that this list covers the full scope of intended uses in the complete set of papers. However, if by reading the full paper, you do find an intended use that is not mentioned in the short list, do specify it in the "other intended uses" field.

Option 'monitoring wild animal numbers' means counting wild animals, make wild animal population estimates/census, detect wild animal presence in certain areas, etc.

Option 'influencing wild animal numbers' is about wild animal population control, protecting threatened wild animals, wild animal translocation, reintroduce and/or prevent extinction of wild animals, etc.

Option 'monitoring wild animal behaviour' means tracking the movements of wild animals and/or their (social) interactions, making habitat/home range estimations, etc.

Option 'influencing wild animal behaviour' means preventing human-wildlife conflict (make sure humans and wild animals are not together in the same place), preventing wild animal-vehicle/machine collision (car, train, aeroplane, turbine, etc.), improving wild animal welfare, etc.

Option 'monitoring wild animal impact' is about epidemic surveillance, quantifying wild animal-vehicle/machine collision (car, train, aeroplane, turbine, etc.), measuring crop damage by wild animals, preventing the spreading of diseases from wild animals to domestic animals and/or humans, etc.

Option 'monitoring human behaviour' is about quantifying human activity in natural areas, monitoring human responses to wild animals or wild animal impacts, etc.

Option 'influencing human behaviour' means preventing human-wildlife conflict (make sure humans and wildlife are not together in the same place), educate humans about wild animal

behaviour, preventing wild animal-vehicle/machine collision (car, train, aeroplane, turbine, etc.), addressing anti-poaching solutions, improve wild animal treatment, etc.

Type of end-user

Note the types of end-users that are explicitly mentioned. However, if the study states that, for example, the intended use is in wildlife management, then the type of end-user “wildlife manager” can be inferred, but do not make educated guesses. Use the field ‘other end-users’ for any specifically mentioned end-users that are not in the short list.

Number of end-users in the field

Note that if the study does not specify the type of end-user it is unlikely that they mention the number of end-users in the field.

Type of human-wildlife interaction

Write down the type of interaction as described in the paper. For example: “dangerous encounters with wildlife”, “wildlife damage to crops/commercial trees/livestock/etc.”, “animal-vehicle collision”, “animal-machine collision”, “wildlife viewing/positive encounters with wildlife”, etc.

Wildlife species

Write the common names of the species as mentioned in the study, preferably including the Latin name in brackets. For example: koala (Phascolarctos cinerus), apaca (Vicugna pacos), etc.

Used digital systems

Option ‘uas’ means Unmanned Aerial System and includes drones, uav (unmanned aerial vehicle), etc.

Option ‘camera trap’ refers to a video/image camera that gets triggered by a sensor, for example based on movement or heat signature.

Option ‘video camera’ refers to a video camera system with continuous recordings as in, the recording is not triggered by external factors, including webcams, CCTV, etc.

Option ‘gnss’ stands for Global Navigation Satellite System and includes GPS, Galileo, Argos and similar technologies that provide position and/or tracking data using satellites.

Option ‘wireless sensor network’ includes IoT networks, and custom-made sensor networks using Raspberry Pi, ESP32, Arduino, etc.

Option ‘acoustic recorder’ includes devices that capture audio, including passive/automatic acoustic recorders and microphones.

Option ‘mobile phone’ includes smartphones and smartphone apps, as well as traditional mobile phone technologies like GSM, SMS, MMS, GPRS, etc.

Option ‘robot’ includes unmanned ground vehicles and automated mobile devices.

Option ‘repellent’ includes devices that, based on data, activate mechanisms like light flashes and sound effects to deter wild animals from entering certain areas.

Option ‘animal borne sensor’ refers to active sensors in collars, mounted devices, surgically inserted sensors, etc. that the wild animal carries around. For example: heartbeat sensor, accelerometer, light intensity, mercury tip, etc.

Option ‘tag’ includes RFID, VHF, radio tags, etc. that the animal carries around but need to be read before data is available.

Option ‘virtual fence’ includes subterranean fibre-optic intrusion detection systems and similar technologies.

Other digital systems

Write down other systems that were used in the study that comply with the definition of digital system in the glossary but were not available in the short list of ‘used digital systems’.

Mentions co-creation with end-users

Mark whether the study mentions that the digital system was created and/or improved using input from the end-users. Possibly because it mentions requirement analysis, end-user trials, feedback questionnaires, etc. (0) no, (1) yes.

Mentions transformational potential

Mark whether the study mentions “transform*”, “transition*” or “change” in relation to the objectives.

Latitude & Longitude

If you do have a location name, but latitude and longitude are not mentioned in the study. Please search for the location in Google Maps and take the latitude and longitude from the result. If you also have no location name or cannot reasonably get a latitude and longitude, leave them empty (do not write 0,0 as that is a valid location somewhere south of Ghana).

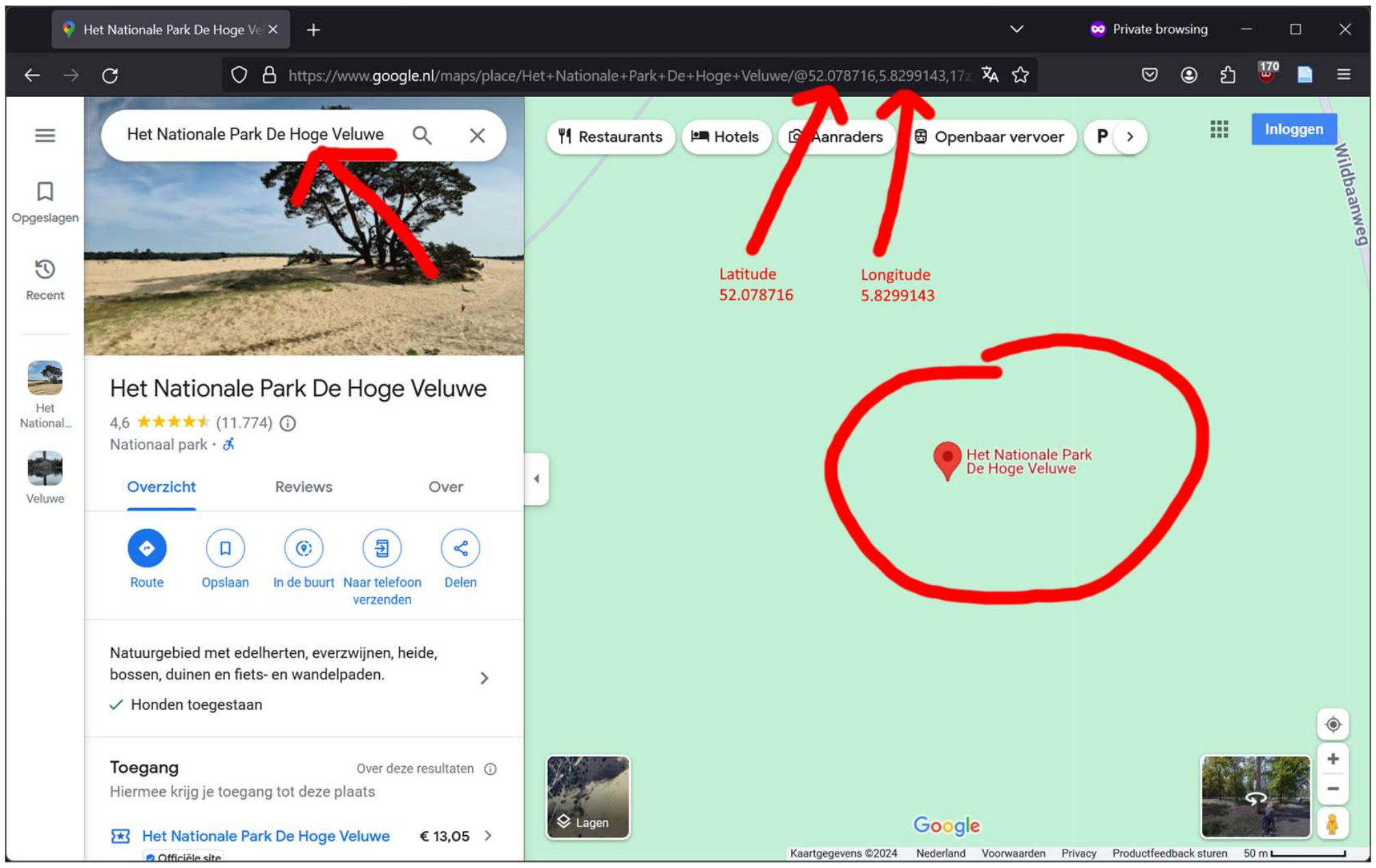

Size of study area

Please specify in km^2. Note that 1 hectare == 0.01 km^2, and 1 km^2 == 100 hectare.

Study duration

Convert the approximate full duration of the study, regardless of pauses or interruptions, to either minutes, days or months and provide an integer. For example: “6 months”, or “15 minutes”. Do not make combinations like “3 months and 15 days” => “106 days”.

Doubt or Issue

Please mark “transformation score doubt” or “digitalization score doubt” with 1 if you experienced doubt when scoring the Transformation/Digitalization metric. Write down a note for yourself in the “issue” column so you can explain your doubt later in a calibration session.

REFERENCES

Abson, D. J., Fischer, J., Leventon, J., Newig, J., Schomerus, T., Vilsmaier, U., von Wehrden, H., Abernethy, P., Ives, C. D., Jager, N. W., & Lang, D. J. (2017). Leverage points for sustainability transformation. *Ambio*, *46*(1), 30–39. https://doi.org/10.1007/s13280-016-0800-y

Bolton, W. (2015). *Programmable logic controllers* (6th edition). Elsevier Newnes.

Dally, W. J., & Poulton, J. W. (1998). *Digital Systems Engineering* (1st ed.). Cambridge University Press. https://doi.org/10.1017/CBO9781139166980

Decker, D. J., Riley, S. J., & Siemer, W. F. (2012). *Human dimensions of wildlife management* (2nd ed). Johns Hopkins University Press.

Raghupathi, W., & Raghupathi, V. (2021). Contemporary Business Analytics: An Overview. *Data*, *6*(8), 86. https://doi.org/10.3390/data6080086

Raj, A. J., & Lal, S. B. (2013). *FORESTRY PRINCIPLES AND APPLICATIONS* (1–1 online resource). SCIENTIFIC PUBLISHER (IND.). http://search.ebscohost.com/login.aspx?direct=true&scope=site&db=nlebk&db=nlabk&AN=1466600
