# Supplementary material for: Transformative potential of digital systems for promoting human-wildlife coexistence: A systematic literature review
Source: Ambio. 2026 May 21;55(12):2836–55. doi: 10.1007/s13280-026-02399-7 (PMC13731620; doi:10.1007/s13280-026-02399-7)
Supplement: Supplementary file 2 [file 13280_2026_2399_MOESM3_ESM.pdf]

Transformative potential of digital systems for promoting human-wildlife coexistence: A systematic literature review

Ambio

Bas S.H.T. Michielsen, Gerard Schouten, Joris P.G. Cromsigt, Remco C. Veltkamp, Irma Arts, Jitse Bijlmakers, Magali Frauendorf, Tim R. Hofmeester, Amy Newsom, Minh-Xuân A. Truong, Helen Verploegen, Els Weinans, Wenjin Yu, Silja Zimmermann, Ine Dorresteijn

Thematic analysis of articles scoring highest on Transformative Potential.

Bas S.H.T. Michielsen
Copernicus Institute of Sustainable Development, Utrecht University, Utrecht, Netherlands
School of ICT, Fontys University of Applied Sciences, Eindhoven, Netherlands
Information and Computing Sciences, Utrecht University, Utrecht, Netherlands
hello@bshtmichielsen.eu

Selected paragraphs and summaries

The six studies were re-read and paragraphs were extracted. Then, for each paragraph a one-sentence summary was created to initiate the coding process.

Citizen science and machine learning: Interdisciplinary approach to non-invasively monitoring a northern marine ecosystem

Modern threats to species and habitats demand multifaceted and collaborative solutions that engage a diverse set of stakeholders and expertise. Given the complexities of many conservation challenges, several authors have proposed solutions that incorporate multiple disciplines, collaborative frameworks, and diverse perspectives to find solutions that fit each issue (Meffe and Viederman, 1995; Naiman, 1999; Dick et al., 2016).

Summary: The importance of interdisciplinary and collaborative approaches in conservation efforts.

Monitoring is an essential part of many successful conservation programs as it allows for adaptive strategies and rapid detection of changes. However, monitoring often receives limited support after the initial conservation action and can be difficult to prioritize when a species or habitat is not currently facing a threat.

Summary: The need for wildlife management to adapt to rapid changes.

Citizen science has been recognized for increasing the capacity of monitoring projects (Chandler et al., 2017). Citizen science, also referred to as community science or participatory science, involves participants contributing to the scientific process through observations, indexing, and even analyzing data (Conrad and Hilchey, 2011). The benefits of citizen science are multifold for both researchers and participants. Harnessing the efforts of volunteers allows researchers to expand their data processing capabilities and can provide data resources for researching and developing machine learning tools (Swanson et al., 2015; Willi et al., 2019; Anton et al., 2021). Additionally, citizen science allows volunteers to meaningfully contribute to scientific endeavors, aiding in increasing scientific literacy and trust in scientific processes (Tulloch et al., 2013).

Summary: The value of citizen science in increasing the capacity of monitoring projects and promoting scientific literacy

Rapid improvement in passive monitoring and data storage technology have also contributed to monitoring efforts by dramatically increasing the size and quality of conservation datasets. Additionally, technological advances have expanded the variety and availability of non- or minimally invasive means of monitoring (Marvin et al., 2016; Stephenson, 2018). There are a growing number of examples where passive acoustic monitoring (Wrege et al., 2017; Wijers et al., 2019), satellite or remote sensing (Luque et al., 2018; Ashutosh and Roy, 2021), and drone monitoring (Burke et al., 2019; Lopez and MuleroPazmany, 2019; Harasyn et al., 2022) are used to increase monitoring effort while remaining cost-effective and minimally invasive to the study species.

Summary: The role of technology, such as machine learning and passive monitoring, in enhancing conservation projects.

Machine learning, a subset of the broader term of artificial intelligence, is an emerging tool that can address the challenges of processing large datasets for conservation projects (Lamba et al., 2019). Deep learning models, a part of machine learning, and in particular convolutional neural networks (CNN), are able to automatically learn useful feature representations at various levels of pixel organization (Krizhevsky et al., 2017).

Summary: The role of machine learning in processing large data sets.

Here, we describe a successful multi-year interdisciplinary, collaborative project for monitoring beluga whales (Delphinapterus leucas) and their marine ecosystem in the Churchill River estuary near Churchill, Manitoba, Canada. We use the terms “interdisciplinary” and “collaborative” intentionally, as our study integrates multiple disciplines to reach a cohesive goal and involves contributions from several stakeholders. This interdisciplinary approach requires collaboration with environmental organizations, biologists and wildlife professionals, research scientists, educators, and community members.

Summary: Defining cohesive goals supported by multiple stakeholders.

The setup includes an underwater camera capturing video footage below the surface, a hydrophone which records beluga vocalizations, and microphones on-board for the vessel operator and onboard guests to commentate during the tour.

Summary: Using different sensors types, such as video and audio, to gather richer information.

We collected images to train the deep learning model from the “General Photo Classification” workflow. Each photo was seen by 10 participants. A curated, balanced set of 12,678 images where 100% of participants agreed on presence or absence of beluga was selected to train and test the model. We used cross-validation to evaluate the generalization of our trained models.

Summary: Use expert curated data as high quality machine learning training data.

Additionally, we applied an attention mechanism to help the model focus on critical and salient regions most pertinent to the object of interest (Jetley et al., 2018). We can gain insight into where the model focuses when it makes predictions by looking at the attention estimator output of the image frames, paying particular attention to images the network struggles to identify or incorrectly assigned class labels.

Summary: The use of explainable AI to evaluate model quality.

Our results demonstrate the value that an interdisciplinary approach has brought to this project, particularly in the integration of wildlife biology, citizen science, and computer science. In collaborating with project partners and citizen scientists, we were able to collect thousands of images each year to index and classify.

Summary: The benefits of working in interdisciplinary teams and with citizen scientists.

While the CNN models were more accurate than citizen scientists in predicting whether belugas were present, this does not discredit the value of these participants in our project. For instance, although the CNN models performed well in determining the presence or absence of beluga, there are more complicated questions we are addressing on Zooniverse. This includes questions that would be challenging for machine learning models to correctly identify, such as classifying unique markings on beluga. Moreover, interactions between participants and researchers on Zooniverse through forum discussions have led to crucial discoveries and project developments (i.e., new jellyfish species, the previously tagged individual). Finally, the aggregated responses from citizen scientists were necessary for testing and training the computer models. This data integration among disciplines highlights an aspect of this approach that we consider essential to the success of our project. For our project, multiple disciplines have been integrated with each other, leading to the development of a shared framework. Citizen scientist classifications would be necessary for any further model development for this project and, in turn, the output from the models could create more engaging workflows for participants in the future.

Summary: Human and machine cooperation.

As the project grows its reach and research capabilities, there is great potential to continue expanding this network to incorporate disciplines and perspectives. For example, understanding the origins and healing processes of injuries and infections can be highly informative for conservation and management. We have established relationships with veterinarians, facilities with animals in care, and researchers to share images of distinct injuries and markings to learn more about them.

Summary: Community engagement creating feedback loops.

We have described a successful interdisciplinary, collaborative monitoring project focusing on beluga whales and the Churchill River estuary ecosystem. This project has been built by bringing together concepts and tools from multiple disciplines with collaborations from diverse partnerships to produce a comprehensive approach.

Summary: Collaboration from diverse partnerships.

Effects of Vessel Distance and Sex on the Behavior of Endangered Killer Whales

Nature-based tourism, including the viewing of free-ranging and often charismatic wildlife, is a well-established industry in many parts of the world. Human activity, however, can have negative consequences on animals, including subsequent effects on the behavior of individuals, social groups and populations, which is especially concerning for vulnerable or endangered species.

Summary: Human activity negatively impacts animal welfare

Both prey availability and disturbance from vessels and noise are identified risk factors to the Southern Resident population that has shown little recovery since ESA listing (National Marine Fisheries Service [NMFS], 2016; Center for Whale Research1, accessed 30 March 2020).

Summary: Human noise and pollution obstruct whale recovery.

Twenty-three digital acoustic recording tags (Dtags) were temporarily attached to individually identified whales from photo-ID records (Center for Whale Research) using a pole from an inflatable research vessel. As conditions allowed, we recorded focal follow data using two integrated equipment packages, each consisting of a GPS/data collector, a laser range finder, and compass (Giles, 2014). One unit recorded whale data, the other recorded vessel data.

Summary: Using different sensors measuring different aspects.

We used HMMs as a multivariate framework to categorize the subsurface behavior of killer whales. This statistical approach has been widely applied in studies that investigate movement and behavior using animal-borne tags that yield time series data, including those on cetaceans (Quick et al., 2016; DeRuiter et al., 2017). HMMs identify the most likely unobservable (hidden) state from observable behavior in sequences that follow a first order Markov process. Using output from the HMMs, we used multinomial logistic regression models to ask whether vessel distance or sex affected state occurrence. We treated the estimated state assignment, based on the most likely (Viterbi) state sequence of the best model, as the response variable, and used vessel distance (2level fixed effect) and deployment number (random effect) as predictors.

Summary: The fusion of different models to make estimations.

We found that vessel distance affected state occurrence and time spent in each state (Figure 4). Overall, whales were less likely to be in states 2–4 (foraging states) than in state 1 (traveling/respiratory, a non-foraging state), and importantly, state 2–4 had a higher occurrence when vessels were far, with the biggest effect for state 2 (deep forage). Furthermore, when we considered time spent in each state, we found that both females and males spent less time in state 3 (acoustic search) and 4 (intermediate dives), and substantially more time in state 2 (deep forage, including prey capture) when vessels were far compared to when vessels were close (Figure 4). These findings suggest that deep foraging opportunities can be enhanced when vessels give whales, especially females, more space.

Summary: The use of technology and statistical models to monitor and understand the impact of human activity on wildlife.

Specifically, females were more likely to transition to a nonforaging (travel/respiratory) state when vessels had an average distance less than 400 yd. A female’s decision to forego foraging in the presence of close vessels could hinder her ability to meet energetic requirements to support reproductive efforts, including fetal growth in pregnancy and lactation costs after calving. This is particularly concerning in an endangered mammalian population because recovery cannot occur without successful reproductive outcomes among breeding individuals, particularly in long-lived females with birthing intervals of 3–7 years (Olesiuk et al., 2005). Our findings, suggesting that female killer whales are at greater risk

from close vessel approaches than males, can inform future management decisions seeking to preserve foraging opportunities and enhance recovery efforts in endangered populations.

Summary: The use of data and statistical analysis to inform management decisions and conservation efforts.

The datasets presented in this study can be found in online repositories. The names of the repository/repositories and accession number(s) can be found below: The NWFSC Public Access to Research Results (PARR)-Data Inventory.

Summary: Data is shared in a FAIR compliant way.

Dark roads aid movement but increase mortality of a generalist herbivore in the American Southwest

The expansion of human populations and associated infrastructure development has reduced and degraded wildlife habitat and increased threats to survival for many wide-ranging wildlife species (Bencin et al., 2019; Forman & Alexander, 1998; Saunders et al., 1991). Roads in particular are linked with numerous negative effects on mammals (Fahrig & Rytwinski, 2009), such as fragmenting habitats (Fraser et al., 2019), restricting movement (Forman & Deblinger, 2000; Schwab & Zandbergen, 2011), and directly causing mortality through vehicle collisions (Bencin et al., 2019; Neumann et al., 2012; Zeller et al., 2018).

Summary: Roads are linked with numerous negative effects on wildlife.

To help fill this gap, we examine the influence of environmental and anthropogenic factors, including artificial nightlight levels, on road crossing behaviors and collision risk of mule deer (Odocoileus hemionus) near Salt Lake City, UT.

Summary: The effect of anthropogenic factors of animal welfare.

Here we used 7 years of mule deer GPS collar movement data (n = 67 individuals) to understand their road crossing behaviors. By linking road crossing behavior with specific landscape, environmental, and road factors, including the novel factor artificial nightlight, our analysis can inform management interventions such as spatially and temporally targeted placement of fences, signage, nightlights, and wildlife crossing structures. Additionally, we use road mortality data to determine whether common factors surround road crossing and mortality locations.

Summary: Using data to inform wildlife management decisions.

We used GPS data from collared mule deer and spatially explicit mule deer road mortality data to analyze road crossing and road mortality site characteristics in the Oquirrh Mountains and greater Salt Lake City area (Figure 1). By aggregating information on a variety of covariates (Table 1) within three different biologically informed buffer zones around the road network (buffer scales: 20, 55, and 573 m), we were able to explore the relationship between the covariates and mule deer crossing probability, intensity of use, and mortality probability.

Summary: Combining models and aggregating information from different sources to optimize prediction quality.

We hypothesized that a variety of landscape, land cover, anthropogenic factors, and road characteristics could influence mule deer road crossing behavior (Table 1). Spatially explicit artificial nightlight estimates were extracted from NASA's Black Marble product.

Summary: Different landscapes and human factors impact wildlife behaviour.

Estimates of 2010 housing density, at a sub-census block unit (100 m2) (National Park Service, 2010), were modeled based on the United States Census Bureau (Theobald, 2005). Road density for our study area was calculated from the USGS National Transportation Dataset shapefile (U.S. Geological Survey, 2021). To do so, we created a blank 50-m raster grid and any cell intersecting a road line segment was given a value of 1, otherwise a 0 for those cells not intersecting a road. To convert this to a measure of road density, the raster was aggregated to 1 km2 such that each cell represented the proportion of each 1-km2 cell with a road segment present. Speed limit information, maintained by UDOT, was included as part of the Road Centerlines dataset obtained from the Utah Geospatial Resource Center (2021). We were therefore able to associate speed limit (in miles per hour) with each road segment. Road segment length was also included in our analysis as a control variable. Bare Earth elevation data were obtained from USGS in raster format as a digital elevation model (DEM) with a 30-m spatial resolution. The terrain roughness index was then derived by taking the mean of the absolute differences between the elevation value of a cell and the value of its eight neighboring cells using the function "terrain" in package "raster" (Hijmans, 2021). Snow cover data, defined as the percentage of each 500-m cell covered by snow in an 8-day period, were obtained from the MODIS/Terra Snow Cover 8-Day L3 Global 500-m SIN Grid (MOD10A2; from the NASA National Snow and Ice Data Center; Hall & Riggs, 2016). We created a year-specific winter composite (16 October–15 April) by taking the mean of snow cover values for each cell. We estimated vegetative greenness using the normalized difference vegetation index (NDVI) derived from MODIS (MOD13Q1) generated every 16 days at 250-m resolution (Didan, 2015). From these data we created year-specific mean NDVI seasonal composites (for summer and winter, as defined above). Land cover data at a 30-m spatial resolution were obtained from the National Land Cover Dataset (NLCD) (U.S. Geological Survey, 2016) and aggregated into five categories: forest (values 41–45), shrub/scrub (values 51 and 52),

developed/urban (values 21–24), agriculture (values 81 and 82), and open/natural (includes herbaceous, open water, and other “open” land types; values 11, 31, 71, 72, 90, and 95) based on ecological significance, horizontal vegetative thickness, and overall impacts to visibility that may influence deer road crossing decisions. We developed two different sets of seasonal regression models for each time of day (day, night, and crepuscular) to examine mule deer road crossing behavior. We used mule deer road mortality data from 2011 to 2018 from the State of Utah Wildlife-Vehicle Collision (WVC) Data Collector repository (Figure 1) to model mortality probability among road segments using the same set of landscape factors (Table 1). When an animal carcass on a roadway is reported to or found by state contractors, they record the species, estimated age class, and GPS coordinate location of the animal in addition to other ancillary information, which is aggregated in the WVC repository.

Summary: Combining data from different sources.

We investigated how artificial nightlight—a pervasive and expanding environmental pollutant—affects patterns of road crossings and mortalities for mule deer.

Summary: Environment pollutants affect wildlife mortality.

Our findings add support to the need for collision mitigation and conservation interventions. Managers could target roadways for mitigation that see both high mortality and high use, such as roads in the winter ranges.

Summary: There is a need for collision mitigation.

Data used in the final models (Frank et al., 2023) are available from Figshare: https://doi.org/10.6084/m9.figshare. 22310314.v1. Novel code is available from Zenodo: https:// doi.org/10.5281/zenodo.7850929.

Summary: Data is shared in a FAIR compliant way.

Mitigating human–wildlife conflict and monitoring endangered tigers using a real-time camera-based alert system

The rebounding of wild tiger (Panthera tigris) populations in India and Nepal has been one of the great conservation success stories of the first quarter of the twenty-first century. Although tigers are still endangered, from a rapidly declining global tiger population 20 years ago, global estimates have increased to approximately 4500 individuals (Dinerstein et al. 2007, Goodrich et al. 2022). Tigers have recovered in large part because of the efforts of governments in tiger-range countries and nonprofit and nongovernmental organizations. The rebound was accelerated by the St. Petersburg Declaration on Tiger Conservation, crafted in 2010 to conserve vital tiger habitat, abate rampant poaching, and target a doubling of the wild population by 2022 (Global Tiger Initiative Secretariat 2011, Global Tiger Forum 2019).

Summary: Conservation success due to government and nongovernmental organizations working together.

Simultaneously, although tigers' numbers have increased, human populations have expanded in the vicinity of tiger reserves, leading to inevitable encounters and conflict. Human populations surrounding nearly all internationally recognized Tiger conservation landscapes (TCLs; Dinerstein et al. 2007) have also grown by over 19.5 million people from 2000 to 2020 (figure 1a, supplemental table S1). The expansion of human development into tiger habitat exacerbates the threats of retaliatory killing, poaching, prey depletion, and habitat degradation for tigers and injury or death for livestock and people (Miller et al. 2016, Jhala et al. 2021). The renewed vision for tiger conservation, following the second Tiger Summit in Vladivostok, Russia, in 2022, demonstrated that there is a continued commitment to a landscapescale management approach that ensures the sustained population growth of this globally imperiled species while sustaining human livelihoods near tiger habitat. These twin goals will require further expansion of protected areas within the world's TCLs and the establishment of corridors linking protected areas that, on their own, remain too small to sustain long-term viable tiger populations without allowing for tiger dispersal and permitting gene flow among reserves (Dinerstein et al. 2006, Wikramanayake et al. 2011, Joshi et al. 2016).

Summary: The population growth of both wildlife and humans lead to inevitable encounters.

In addition, there is increasing recognition of the permanent occupancy of tigers outside tiger reserves. In India, which contains approximately 66% of the world's wild tiger population, approximately 35% of this increasing population permanently resides outside designated tiger reserves (Jhala et al. 2019). The dispersal requirements of the species dictate that individual tigers will increasingly use the interstitial landscapes outside protected areas, therefore occupying habitat highly disturbed by humans (Sunquist and Sunquist 2002).

Summary: Wildlife moves out of their designated areas into habits disturbed by humans.

Human–tiger conflict can take several forms, including the killing of livestock and attacks on people by tigers (Dhanwatey et al. 2013, Karanth et al. 2013) and the retaliatory killing of tigers by angered community members (Inskip and Zimmermann 2009). The resulting human and tiger casualties could reverse wild tiger population gains while further threatening support for conservation among community members in and around TCLs (Goodrich 2010, Letro and Fischer 2020). With forest protection staff spread so thinly across large landscapes (Appleton et al. 2022), new technology that can provide real-time data notifications for conservation personnel could be a massive aid in preventing poaching or conflict before it occurs or afterward for quickly identifying poaching suspects (Hahn et al. 2022).

Summary: The need for technology to help prevent human-wildlife conflicts.

A solution that could provide real-time information on tiger and poacher activity across a landscape would constitute a foundational shift in the ability for wildlife managers and community members to abate conflict and intercede quickly in poaching to better protect one of the world's most imperiled species.

Summary: Technology can constitute a foundational shift in wildlife manager abilities.

In addition, because shared landscapes outside of protected areas in India currently represent a vital part of tiger and other large carnivores' remaining habitat, the viability of these populations may increasingly rely on managing for coexistence with humans, especially where protected area expansion is unlikely (Inskip and Zimmermann 2009, Carter and Linnell 2016). Therefore, conservation solutions that can reduce communications barriers between conservation managers and local communities to stop poaching and prevent human–wildlife conflict are among the most urgent needs in tiger conservation and wildlife management globally.

Summary: Wildlife management strategies need to increasingly rely on managing for coexistence with humans.

Technology is newly emerging to connect authorities with rapidly obtained management data that could be used to resolve conflict. Methods to use AI to identify species combined with communications networks to detect endangered species and alert managers have been tested such as for elephants in Gabon (Whytock et al. 2023). But, as of yet, no such system has used AI on-theedge computing to filter data notifications to just species of interest nor been tested for wild tigers or had its capabilities tested for a large predator in peopled mosaics such as outside tiger reserves.

Summary: The need for technology that can rapidly provide wildlife management data.

Successful deployment of this technology will be aided by the adoption by field personnel and, depending on the scenario, assistance by local community members. We conducted two different education modules for the forest department divisions of the Kanha–Pench corridor and one module for villagers. The purpose of the module for villagers was to educate and produce a dialogue with local community members directly affected by tiger conflict and to identify a community steward to employ to assist with maintenance of the camera-alert system. This module presented a general framework of stewardship and engagement to strengthen community capacity, develop understanding in efforts toward mitigating human–carnivore conflict through targeted education material, foster stewardship and empowerment through local community participation.

Summary: The need to involve the community with education.

The use of real-time systems such as TrailGuard AI is a major shift from the generally passive use of camera trap data to the active use of AI-filtered camera trap notifications. Compared with camera trap studies in which the design, collection, and analysis of photos can take place siloed within a research group or management unit, the use of these systems' full potential requires the active involvement of field staff that have day-today interactions with the poaching threat and human–wildlife conflict. There must be buy-in from these personnel because the utility of real-time data for prevention of poaching or conflict could be lost without rapid action by the proper authorities. Engaged collaborations will be important to assist with initial training on the deployment of the camera system and lessons learned in using the data notifications. Real-time camera data also opens a new realm of socioecological research questions, whose answers will aid the efficacy of conservation efforts using these technologies. These questions could include when and where poaching incidents decrease after system deployment, an evaluation of timely actions by resource managers related to detection notifications, and the subsequent response of local residents to intelligence about the presence of conflict-prone species. In areas close to human habitation where the goal is the prevention of human–wildlife conflict, further research and management actions should be coupled with initiatives that educate people on the importance of wild tigers and promote equitable cooperation between local communities and frontline staff (Johnson et al. 2018, Ardoin et al. 2020).

Summary: The importance of engaged collaborations.

Finally, the application of systems such as TrailGuard AI goes well beyond the conservation of tigers to other imperiled large mammal species for which AI detector models have been developed by CVEDIA (see supplement S1), such as snow leopards (Panthera uncia), Asiatic elephants, various species of bears, and gray wolves (Canis lupus). The camera-alert system has shown its utility in protecting African savanna elephants

(Loxodonta africana) by alerting rangers and the rangers subsequently apprehending multiple groups of poachers in Serengeti National Park and other protected areas of eastern Africa (Dinerstein 2018). Used correctly and at the appropriate density for the unique situation, this camera-alert system has the potential to improve direct, timely intervention for wildlife conservation.

Summary: Advanced technology can help in a variety of different wildlife management situations.

Evaluating the efficacy of a landscape scale feral cat control program using camera traps and occupancy models

Many species of Australian native mammals have experienced significant range declines or extinction in the arid zone since European settlement, and many species of birds have suffered similar declines over the same time period. One of the often cited causes for this decline in native species is predation by introduced predators, in particular the feral cat (Felis catus) and red fox (Vulpes vulpes).

Summary: Wildlife conservation is hampered by previous introduction of introduced predators.

Baiting is recognised as the most effective method for controlling feral cats in particular when there is minimal risk posed to non-target species. Effective and cost-efficient control of feral cats in large reserves, which often have restricted access, can only be achieved through aerial baiting campaigns. The expansiveness of Pilbara landscapes necessitates landscape-scale abatement of introduced predators, as small-scale localised control programs are unlikely to achieve conservation objectives.

Summary: Baiting is a recognised wildlife population control method.

Monitoring the abundance of feral cats, like many mammalian carnivores, is difficult because they occur at low densities, have large home ranges and tend to be secretive and cryptic. Capture-recapture studies to estimate abundance are usually impractical as cats are difficult to trap, leading to low capture rates and recapture probabilities. Consequently, most monitoring schemes rely on indices of abundance.

Summary: Monitoring cryptic animals is notoriously difficult, which makes population control difficult.

Camera trap studies are useful in providing information on feral cat presence/absence but in many cases individuals cannot be easily distinguished with certainty. At a rudimentary level, presence/absence data from camera trap sites can be used to provide indices of relative activity. Raw detection rates (i.e. total number of sites where cats were detected/number of sites) are naïve estimates of occupancy that do not account for probability of detection. If detection probabilities are determined, estimates of occupancy can be derived from presence/ absence data. Occupancy is often used as a metric for estimating various species' occurrence and is a function of abundance as it concerns the probability of a particular animal being at a given site. Rather than giving an estimate of population size, occupancy models calculate the probability of a site being occupied and provide a repeatable and objective means of evaluating the success of population knockdown in a structured decision-making framework. This study aimed to determine the usefulness of camera traps for establishing feral cat baiting efficacy using Eradicat baits in the Fortescue Marsh, Western Australia. Repeat camera survey data were used to establish if site occupancy models could be reliably used to demonstrate baiting effectiveness.

Summary: Camera traps provide naïve estimates of wildlife abundance.

In 2014, mortality of six of the seven radio-collared cats was attributed to bait consumption, and occurred within 23 days of baiting. The seventh collared cat in 2014 was euthanized, having survived the baiting. Mortality of four of eleven collared cats was attributed to baiting in 2015 and nine of eleven in 2016. The surviving cat from 2014, and six out of seven bait survivors from 2015, were euthanized one month post-baiting. Extrapolating the mortality figures to the larger population, 85% of feral cats in the baited area died as a result of baiting in 2014, 36% in 2015 and 82% in 2016.

Summary: Baiting success can be estimated using wildlife collars.

The large home ranges, cryptic nature and general low densities of feral cats present significant challenges for managers trying to monitor effectiveness of control programs17,19. The use of cameras provides an alternative to trapping, track counts and direct bait uptake to measure changes in feral cat populations pre- and post-treatment. This study showed that infrared cameras can be utilised to monitor the efficacy of landscape-scale feral cat control. The results of this study indicate that baiting, while not 100% effective, can have a significant impact on feral cat activity. The potential benefits of an effective and broad-scale feral cat control program to the biodiversity values of the Fortescue Marsh area are significant; with the marsh containing habitat for a number of threatened mammals and ground-nesting and migratory birds. Landscape-scale baiting programs are the only currently available mechanism to implement control in areas of this size, but it is imperative that the success or failure of the program can be effectively gauged.

Summary: Broad-scale population control programmes are essential to protect biodiversity values.

Our study also supports the utilisation of camera traps for monitoring the response of a feral cat population to a baiting program over multiple years. Over the three years (2014–2016) where feral cats were radio-tracked only one radio collared cat was detected by a camera, indicating that the cameras only detect a proportion of the population; which in this case provided sufficient data for robust occupancy modelling. Improving the probability of detecting the target species is highly desirable. This becomes particularly relevant after sustained removal programs and an expected decline in the target animal following successful control programs. Extending the period of camera deployment could overcome this problem; however, the longer the monitoring period the greater the probability that the assumption of the system being 'closed' will be violated. This could be overcome by increasing camera numbers across a larger treatment area, or improving the attractiveness of the lures to attract more animals in the system; however, the practicalities of resourcing and managing large numbers of cameras may make this impractical for conservation managers.

Summary: Combining data from different sources increases information quality

The low detection of collared cats on cameras also indicates that cat detection by cameras is a low probability event, which may have implications for monitoring of feral cat populations at low densities during eradication programs. For this reason, the spatial model employed was considered a more reliable estimate of occupancy as this model accounts for any spatial autocorrelation between cameras.

Summary: The need for combining occupancy models with other data sources.

In our study occupancy models using camera trap data have clearly demonstrated the efficacy of baiting programs over large areas in successive years. The use of these easily replicated techniques for measuring success or failure of such programs is of enormous value to conservation managers.

Summary: Occupancy modelling is effective in gauging bating success.

Acoustic Monitoring Confirms Significant Poaching Pressure of European Turtle Doves (Streptopelia turtur) during Spring Migration across the Ionian Islands, Greece

There are over 120 Afro-Palearctic migrant bird species which fly northwards each spring to breed in Europe, returning to sub-Saharan Africa to overwinter [1]. An assessment of 38 of them from 1980 to 2009 by the Pan-European Common Bird Monitoring Scheme (PECBMS) showed 71%

having a declining population trend that was often more pronounced than that of the resident or short-distance migrants [2]. Due to the migrant species' reliance during their annual cycle on resources extending across multiple ranges of countries, they are exposed to a diverse array of anthropogenic pressures, including habitat degradation due to land-use, and climate change and direct mortality, including over-harvesting [3].

Summary: Migrant birds are exposed to a diverse array of anthropogenic pressures.

In part due to the predictable timing of the migration of entire populations twice each year, migrant species are especially vulnerable to illegal killing or trapping at sites across the Mediterranean where the birds stop-over to rest and/or refuel along their main migratory routes [4]. Recognizing the conservation importance of this threat, the Convention on the Conservation of Migratory Species of Wild Animals adopted in 2014 a resolution and established a task force to address bird crime in the region (”Intergovernmental Task Force to address illegal killing, taking and trade of migratory birds in the Mediterranean” [5]).

Summary: The need for governmental support of conservation efforts.

To achieve these objectives, first the current population decline of turtle doves must be halted. The ISSAP identifies illegal killing as a one of the critical threats that turtle doves face that needs to be eradicated in the European Union (EU) and reduced in other range countries. Evaluating the scale of illegal killings is an essential first step towards this goal according to the ISSAP, along with enhancing the enforcement of hunting regulations especially at hot-spots of illegal turtle dove killings [8].

Summary: The need for monitoring of illegal killing of wildlife.

Passive acoustic monitoring (PAM) has gained increased recognition in recent years as an efficient method for estimating with high spatial and temporal resolution gun hunting pressure across large areas [13,14]. Typically, a grid of semi-autonomous acoustic sensors are deployed across a landscape to record continuously the ambient sound (soundscape) for extended periods of time (weeks or months), with the data subsequently being scanned either manually or by using detection algorithms to locate the events of interest (i.e., the time and date of gunshots per sensor).

Summary: Technologies, like passive acoustic recorders, are an efficient way of detecting illegal killings of wildlife

The local hunters consider the poaching of turtle doves during spring migration to be such a local custom and continue the illegal hunts despite it being banned in Greece since 1985. Originally, the main motivation for poaching turtle doves was self-consumption and gifting surplus birds to friends and family, but it has progressively turned into a mean of demonstrating wealth and social status [10].

Summary: The importance of cultural impacts and worldviews.

The collected data were used to (a) confirm the feasibility of monitoring gun hunting activity in the region using PAM methods, (b) assess the performance (recall rate) of the algorithm used to detect gunshots, and (c) refine the recording schedule in the following years.

Summary: Ensuring high quality data.

Even though the ring recovery data provide only a rough description of the breeding populations impacted upon by the Ionian Islands' spring poaching, the illegal turtle dove harvest that we reported in 2021 exceeded 3.7% of the combined breeding pairs of these 13 countries—and it was larger than the entire breeding population of four of them (Table S2). Incurring such losses at only a fraction of the turtle dove's eastern spring migration flyway no doubt hinders, or possibly even cancels, the conservation measures taken at these countries to reverse the existing decreasing population trends.

Summary: The countereffects of illegal killings on conservation efforts.

Regardless, the passive acoustic monitoring (PAM) data presented here serve as a baseline of the current status quo, enabling the relevant authorities to assess with unprecedented spatial and temporal resolution the effectiveness of future conservation measures. The striking difference between the PAM and the patrol-based findings on the level of poaching activity in 2021 highlights the urgency and importance of reconsidering the current anti-poaching strategy. Clearly, when a patrol fails to report any poaching activity in the Keri peninsula (12 km2), Zakynthos, on a day that our acoustic sensor—located meters off a main, public forest road—recorded 578 gunshots (927 adjusted for the detector's recall rate; 13 April 2021; Table S2), there are issues beyond human and financial resource limitations that have to be addressed.

Summary: Traditional wildlife and conservation management strategies may be ineffective.

Nevertheless, there is the precedence of successfully eliminating turtle dove poaching at a previous hot-spot in the Ionian Islands—the two Strofadia islets (2.5 km2 and 0.1 km2) located 45 km southwest of Zakynthos (Figure 1), where spring poaching was rampant until the turn of the

21st century. Poaching ceased after the combined and sustained efforts of park/game guards, and police and coast guard forces, and the personnel of the Holy Metropolis of Zakynthos, to which the Strofadia islets belong. There are lessons from this success case that can be transferred to the rest of the Ionian Islands, starting with the will and commitment for change.

Summary: The need for cooperation of different governmental stakeholders.

In addition, a citizen science project could be developed to collect information on the activity (or equally important inactivity) of known “posta”, at a coarser temporal scale. Such a project would have the additional benefit of engaging the public (e.g., conservation sensitive citizens, tourists, and local hunting club/Hellenic Hunting Confederation members), empowering them to be part of the solution of a local conservation challenge.

Summary: Possible gains from citizen science and community involvement.

We believe that the evidence presented in this study regarding the persistence and scale of the spring migration poaching of turtle doves in the Ionian Islands should be seen as a call for reconsidering turtle dove conservation and management actions in Greece. With sustained political support for the strict enforcement of wildlife laws across the Ionian Islands, we believe that the spring poaching can be eliminated in less than five years.

Summary: The need for political support in wildlife conservation.

Coding

The summary column contains one-sentence summaries of the selected paragraphs of the studies.

Citizen science and machine learning: Interdisciplinary approach to non-invasively monitoring a northern marine ecosystem

Summary	Code
The importance of interdisciplinary and collaborative approaches in conservation efforts.	Interdisciplinary collaboration
The need for wildlife management to adapt to rapid changes.	Adaptive conservation strategies
The value of citizen science in increasing the capacity of monitoring projects and promoting scientific literacy.	Citizen science and community engagement
The role of technology, such as machine learning and passive monitoring, in enhancing conservation projects.	Technology-enhanced conservation
The role of machine learning in processing large data sets.	Technology-enhanced conservation
Defining cohesive goals supported by multiple stakeholders.	Interdisciplinary collaboration
Using different sensors types, such as video and audio, to gather richer information.	Technology-enhanced conservation
Use expert curated data as high quality machine learning training data.	Citizen science and community engagement
The use of explainable AI to evaluate model quality.	Technology-enhanced conservation
The benefits of working in interdisciplinary teams and with citizen scientists.	Interdisciplinary collaboration
Human and machine cooperation.	Human-machine cooperation
Community engagement creating feedback loops.	Citizen science and community engagement
Collaboration from diverse partnerships.	Interdisciplinary collaboration

Effects of Vessel Distance and Sex on the Behavior of Endangered Killer Whales

Summary	Code
Human activity negatively impacts animal welfare	Anthropogenic impacts on wildlife welfare

Human noise and pollution obstruct whale recovery.	Anthropogenic impacts on wildlife welfare
Using different sensors measuring different aspects.	Technology-enhanced conservation
The fusion of different models to make estimations.	Conservation data analysis
The use of technology and statistical models to monitor and understand the impact of human activity on wildlife.	Anthropogenic impacts on wildlife welfare
The use of data and statistical analysis to inform management decisions and conservation efforts.	Informing management decisions
Data is shared in a FAIR compliant way.	Data sharing

Dark roads aid movement but increase mortality of a generalist herbivore in the American Southwest

**Summary**	**Code**
Roads are linked with numerous negative effects on wildlife.	Anthropogenic impacts on wildlife welfare
The effect of anthropogenic factors of animal welfare.	Anthropogenic impacts on wildlife welfare
Using data to inform wildlife management decisions.	Data-driven conservation management
Combining models and aggregating information from different sources to optimize prediction quality.	Combining data sources to obtain new information
Different landscapes and human factors impact wildlife behaviour.	Anthropogenic impacts on wildlife welfare
Combining data from different sources.	Combining data sources to obtain new information
Environment pollutants affect wildlife mortality.	Anthropogenic impacts on wildlife welfare
There is a need for collision mitigation.	Data-driven conservation management
Data is shared in a FAIR compliant way.	Data sharing

Mitigating human–wildlife conflict and monitoring endangered tigers using a real-time camera-based alert system

**Summary**	**Code**

Conservation success due to government and nongovernmental organizations working together.	Collaborative conservation approaches
The population growth of both wildlife and humans lead to inevitable encounters.	Human-wildlife conflict mitigation
Wildlife moves out of their designated areas into habits disturbed by humans.	Human-wildlife conflict mitigation
The need for technology to help prevent human-wildlife conflicts.	Human-wildlife conflict mitigation
Technology can constitute a foundational shift in wildlife manager abilities.	Technology-enabled conservation
Wildlife management strategies need to increasingly rely on managing for coexistence with humans.	Adaptive management for human-animal coexistence
The need for technology that can rapidly provide wildlife management data.	Technology-enabled conservation
The need to involve the community with education.	Community engagement and education
The importance of engaged collaborations.	Collaborative conservation approaches
Advanced technology can help in a variety of different wildlife management situations.	Technology-enabled conservation

Evaluating the efficacy of a landscape scale feral cat control program using camera traps and occupancy models

Summary	Code
Wildlife conservation is hampered by previous introduction of introduced predators.	Anthropogenic impacts on wildlife welfare
Baiting is a recognised wildlife population control method.	Wildlife population control
Monitoring cryptic animals is notoriously difficult, which makes population control difficult.	Wildlife population control
Camera traps provide naïve estimates of wildlife abundance.	Wildlife population control
Baiting success can be estimated using wildlife collars.	Wildlife population control
Broad-scale population control programmes are essential to protect biodiversity values.	Adaptive conservation strategies
Combining data from different sources increases information quality	Technology-enabled management
The need for combining occupancy models with other data sources.	Wildlife population control
Occupancy modelling is effective in gauging bating success.	Adaptive conservation strategies

Acoustic Monitoring Confirms Significant Poaching Pressure of European Turtle Doves (Streptopelia turtur) during Spring Migration across the Ionian Islands, Greece

Summary	Code
Migrant birds are exposed to a diverse array of anthropogenic pressures.	Anthropogenic pressure on wildlife conservation
The need for governmental support of conservation efforts.	Governmental support for conservation efforts
The need for monitoring of illegal killing of wildlife.	Adaptive conservation strategies
Technologies, like passive acoustic recorders, are an efficient way of detecting illegal killings of wildlife	Technology and data-driven conservation.
The importance of cultural impacts and worldviews.	Community engagement and education.
Ensuring high quality data.	Technology and data-driven conservation.
The countereffects of illegal killings on conservation efforts.	Adaptive conservation strategies
Traditional wildlife and conservation management strategies may be ineffective.	Adaptive conservation strategies
The need for cooperation of different governmental stakeholders.	Governmental support for conservation efforts
Possible gains from citizen science and community involvement.	Community engagement and education.
The need for political support in wildlife conservation.	Governmental support for conservation efforts

Themes

Themes were created based on a process of grouping similar codes from the coding process.

Theme	Codes
**Creating trans-/inter-disciplinary groups including IT experts and field experts and citizen scientists.**	Interdisciplinary cooperation, Collaborative conservation approaches
**Addressing (also) the impact of human activity on wildlife.**	Anthropogenic impacts on wildlife welfare, Human-wildlife conflict mitigation, Adaptive management for human-animal coexistence, Adaptive conservation strategies, Anthropogenic pressure on wildlife conservation
**Using of advanced modelling techniques (statistical as well as deep learning and/or machine learning) to take data driven informed decisions.**	Technology-enhanced conservation, Human-machine cooperation, Conservation data analysis, Data-driven conservation management, Technology-enabled conservation, Technology-enabled management, Technology and data-driven conservation
**Integrating data from different sources (databases, sensors, citizen science, etc.) and share data with others.**	Data sharing, Combining data sources to obtain new information
**Engaging and educating governments and communities.**	Citizen science and community engagement, Informing management decisions, Community engagement and education, Governmental support for conservation efforts
