# Supplementary material for: Transformative potential of digital systems for promoting human-wildlife coexistence: A systematic literature review
Source: Ambio. 2026 May 21;55(12):2836–55. doi: 10.1007/s13280-026-02399-7 (PMC13731620; doi:10.1007/s13280-026-02399-7)
Supplement: Supplementary file 3 [file 13280_2026_2399_MOESM2_ESM.html]

analysis


Analysis

Transformative potential of digital systems for promoting human-wildlife coexistence: A systematic literature review  
Ambio  
Bas S.H.T. Michielsen, Gerard Schouten, Joris P.G. Cromsigt, Remco C. Veltkamp,
Irma Arts, Jitse Bijlmakers, Magali Frauendorf, Tim R. Hofmeester, Amy Newsom,
Minh-Xuân A. Truong, Helen Verploegen, Els Weinans, Wenjin Yu, Silja Zimmermann,
Ine Dorresteijn

Bas S.H.T. Michielsen  
Copernicus Institute of Sustainable Development, Utrecht University, Utrecht, Netherlands  
School of ICT, Fontys University of Applied Sciences, Eindhoven, Netherlands  
Information and Computing Sciences, Utrecht University, Utrecht, Netherlands  
hello@bshtmichielsen.eu

In [2]:

```
import geopandas, numpy, os, pandas, platform, seaborn
import matplotlib.pyplot as plt
from typing import Optional, Tuple

seaborn.set_theme()
score1 = "Systemic Depth"
score2 = "Digital Maturity"
score_total = "Transformative Potential"
framework = "Digital Sustainability Transformation Framework"

def draw_barplot(data: pandas.Series, title: str, size: Tuple[int, int] = (6, 3), xlim: Optional[Tuple[float, float]] = None):
    if xlim == None: xlim = (0, data.values.max() * 1.1)
    plt.figure(figsize=size)
    plot = seaborn.barplot(x=data.values, y=data.index, palette="Set2", hue=data.index, legend=False)
    plot.set(title=title, ylabel="", xlim=xlim)
    for index, value in enumerate(data.values):
        plot.text(value, index, " " + str(value), va="center", fontsize=8)
    plt.show()

oceans = geopandas.read_file("shapes/ne_50m_ocean/ne_50m_ocean.shp") # download from: https://www.naturalearthdata.com/http//www.naturalearthdata.com/download/50m/physical/ne_50m_ocean.zip
print("Python", platform.python_version(), "| Pandas", pandas.__version__, "| Seaborn", seaborn.__version__)
```

```
Python 3.12.10 | Pandas 2.2.2 | Seaborn 0.13.2
```

In [3]:

```
df = pandas.read_csv("dataset.csv")
print("The number of studies retrieved:", len(df))
```

```
The number of studies retrieved: 1041
```

# Exclusions¶

In [4]:

```
plot_data = pandas.Series({"EX1":len(df[df["EX1"]==True]),"EX2":len(df[df["EX2"]==True]),"EX3":len(df[df["EX3"]==True]),"EX4":len(df[df["EX4"]==True])})
draw_barplot(plot_data, "Exclusions (n="+str(plot_data.sum())+")")
```

In [5]:

```
df = df[df[score_total].notna()]
print("The number of studies included:", len(df))
```

```
The number of studies included: 524
```

# Transformative Potential¶

In [6]:

```
max_score = 16
plot_data = df[[score1, score2]].astype(int)
plot_data["Count"] = 1
plot_data = plot_data.groupby([score1, score2], as_index=False).count()
plot_data = pandas.pivot_table(plot_data, values="Count", index=score1, columns=score2)
for i in range(max_score):
    name = i +1
    if name not in plot_data.columns:
        plot_data.insert(i, name, [numpy.nan for x in range(len(plot_data))])
for i in range(max_score):
    name = i +1
    if name not in plot_data.index:
        plot_data.loc[name] = [numpy.nan for x in range(max_score)]
plot_data.sort_index(inplace=True)
seaborn.set(rc={"figure.figsize":(8, 8)})
plot = seaborn.heatmap(plot_data, cmap="Blues", annot=True, vmax=35, cbar=None)
plot.set_title(framework, fontsize=16)
plot.invert_yaxis()
```

In [7]:

```
seaborn.boxplot(df[[score1, score2]])
df[[score1, score2]].describe().apply(lambda x: x.apply('{0:.2f}'.format))
```

Out[7]:

|  | Systemic Depth | Digital Maturity |
| --- | --- | --- |
| count | 524.00 | 524.00 |
| mean | 3.23 | 3.80 |
| std | 2.01 | 1.96 |
| min | 1.00 | 1.00 |
| 25% | 2.00 | 2.00 |
| 50% | 2.00 | 4.00 |
| 75% | 4.00 | 5.00 |
| max | 12.00 | 12.00 |

In [8]:

```
skew = df[[score1, score2]].skew()
print("Skewness coefficients:")
skew
```

```
Skewness coefficients:
```

Out[8]:

```
Systemic Depth      1.818145
Digital Maturity    0.703034
dtype: float64
```

In [9]:

```
for score in [score1, score2]:
    s = df[df[score] < df[score].mean()]
    print("Number of studies scoring less than the mean on", score, ":", s.shape[0], "(", s.shape[0] / df.shape[0] * 100,"% )")
```

```
Number of studies scoring less than the mean on Systemic Depth : 376 ( 71.7557251908397 % )
Number of studies scoring less than the mean on Digital Maturity : 256 ( 48.854961832061065 % )
```

In [10]:

```
score1_counts = df[score1].value_counts().reindex(numpy.arange(1, 17))
score2_counts = df[score2].value_counts().reindex(numpy.arange(1, 17))
scores_df = pandas.DataFrame({'Score': numpy.tile(numpy.arange(1, 17), 2), 'Frequency': numpy.concatenate([score1_counts.values, score2_counts.values]), 'Type': [score1] * 16 + [score2] * 16})
plt.figure(figsize=(8, 6))
seaborn.barplot(data=scores_df, x='Score', y='Frequency', hue='Type', palette='Set2')
print("Variance of", score1, ":", df[score1].var(), "|", "Variance of", score2, ":", df[score2].var())
```

```
Variance of Systemic Depth : 4.060090785690307 | Variance of Digital Maturity : 3.8343380088450374
```

In [11]:

```
plot_data = df[["Parameters", "Feedbacks", "Design", "Intent", "Explains LP interactions", "Visualizations", "Statistical modelling", "Machine Learning", "Data Storing & Sharing"]]

plot_data = plot_data.astype(int)

fig, axs = plt.subplots(2,5, figsize=(12, 6), sharey=True)
color_map = {0:"lightgrey", 1:"blue", 2:"blue", 3:"blue", 4:"blue"}
for i, (col, ax) in enumerate(zip(plot_data.columns, axs.flatten())):
    order = [0, 1, 2] if i < 4 else [0, 1, 2, 3, 4] if i > 4 else [0, 1] 
    seaborn.countplot(data=plot_data, x=col, ax=ax, order=order, palette=color_map, hue=col, legend=None)
    total = plot_data[col].count()
    for p in ax.patches:
        height = p.get_height()
        percentage = (height / total) * 100
        ax.text(p.get_x() + p.get_width()/2, height + 40, str(int(height)), ha="center", fontsize=9.5)
        ax.text(p.get_x() + p.get_width()/2, height + 5, f"{percentage:.1f}%", ha="center", fontsize=9.5)
axs[1,4].set_visible(False)
fig.tight_layout()
fig.subplots_adjust(hspace=0.5)
```

# Mentions transformational protential¶

In [12]:

```
plot_data = df.groupby(by="Mentions transformational potential").count()["ID"].rename("Count")
plot_data.index = plot_data.index.map({1: "yes", 0: "no"})
draw_barplot(plot_data, "Mentions transformational potential", (6, 1))
```

# Study areas¶

In [13]:

```
def multi(continent, count):
    result = []
    for _ in range(count):
        result.append(continent[0])
    return result

plot_data = df[["Continent", "Latitude", "Longitude"]].dropna()
plot_data["Continent"] = plot_data["Continent"].str.split(";")
plot_data["Latitude"] = plot_data["Latitude"].str.split(";")
plot_data["Longitude"] = plot_data["Longitude"].str.split(";")
plot_data["Continent"] = plot_data.apply(lambda x: multi(x["Continent"], len(x["Latitude"])) if len(x["Continent"]) == 1 and len(x["Latitude"]) > 1 else x["Continent"] ,axis=1)
print("Number of studies that mention at least one study area:", len(plot_data))
plot_data = plot_data.explode(["Continent", "Latitude", "Longitude"]).reset_index(drop=True)
plot_data["Latitude"] = pandas.to_numeric(plot_data["Latitude"], errors='coerce')
plot_data["Longitude"] = pandas.to_numeric(plot_data["Longitude"], errors='coerce')
fig, ax = plt.subplots(figsize=(12, 12))
oceans.plot(ax=ax, color="blue", alpha=.5)
for _, row in plot_data.iterrows(): plt.scatter(row["Longitude"], row["Latitude"], marker=".", color="red", alpha=.5)
pos = {"Africa" : [-17,-10], "Antarctica" : [-20,-64], "Asia" : [130,20], "Europe" : [-42,45], "North America" : [-177,30], "Oceania" : [110,-47], "South America" : [-127,-35]}
plot_counts = plot_data.groupby(by="Continent").count()["Latitude"].sort_index()
for name, value in plot_counts.items(): plt.text(pos[name][0],pos[name][1], name + " " + str(value), color="white")
plt.xlim(-180, 180); plt.ylim(-90, 90); plt.xlabel("Longitude"); plt.ylabel("Latitude"); plt.grid(visible=False)
plt.show()
```

```
Number of studies that mention at least one study area: 420
```

# Digital System Use¶

In [14]:

```
plot_data = df["Used digital systems"].str.get_dummies(sep=';')
plot_data["Year"] = df["Year"]
totals = plot_data.groupby("Year").sum()
uniques = (totals > 0).sum(axis=1)
totals = totals.sum(axis=1)
yearly = plot_data.groupby("Year").size()
valid_years = yearly[yearly >= 10].index
percentages = plot_data.groupby("Year").mean() * 100 
percentages = percentages[percentages.index.isin(valid_years)]
totals = totals[totals.index.isin(valid_years)]
plot_data = percentages.reset_index().melt(id_vars="Year", var_name="Digital System", value_name="Prevalence (%)").sort_values(by=["Year", "Prevalence (%)"], ascending=False)
palette = seaborn.color_palette("tab20")
palette = palette[::2] + palette[1::2] 
palette = palette[:17]
plt.figure(figsize=(12, 6))
seaborn.lineplot(data=plot_data, x="Year", y="Prevalence (%)", hue="Digital System", marker='o', palette=palette)
plt.legend(title="Digital Systems", bbox_to_anchor=(1.05, 1), loc="upper left")
for i, year in enumerate(totals.index):
    plt.text(year, -7, "n = "+str(totals[year]), ha="center", va="top", fontsize=8)
    plt.text(year, -9, "S = "+str(uniques[year]), ha="center", va="top", fontsize=8)
plt.xlabel("Year", labelpad=28)
plt.xticks(percentages.index); plt.grid(); plt.tight_layout()
```

# Countries¶

In [15]:

```
plot_data_countries = df["Country"].str.get_dummies(sep=';').sum(min_count=1).loc[lambda x: x > 1].sort_index(ascending=True).sort_values(ascending=False, kind='mergesort')
draw_barplot(plot_data_countries, "Countries mentioned more than once (n=" + str(plot_data_countries.sum()) + ")", (6,9))
```

In [16]:

```
countries = df["Country"].str.get_dummies(sep=';').sum(min_count=1).loc[lambda x: x == 1].sort_index()
print("Countries that were mentioned once:\n", ", ".join(list(countries.index)))
```

```
Countries that were mentioned once:
 Argentina, Austria, Bahamas, Chile, Cote D'Ivoire, Croatia, Democratic Republic Of Congo, Egypt, Equatorial Guinea, Ethiopia, Finland, Gabon, Gibraltar, Hawaii, Ireland, Ivory Coast, Korea, Madagascar, Malawi, Mongolia, Mozambique, Nepal, Netherlands, Pakistan, Peru, Republic Of Korea, Saudi Arabia, Scotland, Singapore, Switzerland, Taiwan, Thailand, Trinidad, Zambia
```

# Biomes¶

In [17]:

```
plot_data_biomes = df[["Biome"]].dropna()
plot_data_biomes = plot_data_biomes["Biome"].str.split(";")
plot_data_biomes = plot_data_biomes.explode(["Biome"]).value_counts().sort_values(ascending=False)
draw_barplot(plot_data_biomes, "Number of study locations per biome (n=" + str(plot_data_biomes.sum()) + ")", (6,5))
```

# Digital systems¶

In [18]:

```
plot_data_digital_systems = df["Used digital systems"].str.get_dummies(sep=';').sum().sort_values(ascending=False)
draw_barplot(plot_data_digital_systems, "Frequency of Digital Systems Mentioned (n="+str(plot_data_digital_systems.sum())+")", (5, 6))
```

# Taxonomic classes and studied species¶

In [19]:

```
plot_data_taxonomic_classes = df["Taxonomic classes"].str.get_dummies(sep=';').sum().sort_values(ascending=False)
draw_barplot(plot_data_taxonomic_classes, "Number of studies per taxonomic class (n="+str(plot_data_taxonomic_classes.sum())+")", (6,3))
```

In [20]:

```
n = 3

def extract_latin_names(species):
    if pandas.isna(species): return ""
    latin_names = []
    for part in species.split(';'):
        name = part.strip()[part.index('(')+1:part.index(')')] if '(' in part and ')' in part else part
        latin_names.append(name)
    return ";".join(latin_names)

plot_data = df
plot_data["Wildlife species"] = plot_data["Wildlife species"].apply(lambda x: extract_latin_names(x))
plot_data = plot_data["Wildlife species"].str.lower().str.get_dummies(sep=';').sum().loc[lambda x : x >= n].sort_index(ascending=True).sort_values(ascending=False, kind='mergesort')
draw_barplot(plot_data, "Number of studies per species (>="+str(n)+")", (6, 11))
```

# Intended Use¶

In [21]:

```
new_index = ['Monitoring Wild Animal Numbers', 'Influencing Wild Animal Numbers','Monitoring Wild Animal Behaviour','Influencing Wild Animal Behaviour', 'Monitoring Wild Animal Impact', 'Monitoring Human Behaviour', 'Influencing Human Behaviour']
plot_data_intended_uses = df["Intended uses"].str.get_dummies(sep=';').sum().reindex(new_index)
draw_barplot(plot_data_intended_uses, "Number of studies per mentioned intended use (n="+str(plot_data_intended_uses.sum())+")")
```

# Type of end-user and co-creation¶

In [22]:

```
plot_data_end_users = df.groupby(by="Type of end-user")["ID"].count().rename("")
draw_barplot(plot_data_end_users, "Mentioned end-users (n="+str(plot_data_end_users.sum())+")", (6,3))
```

In [23]:

```
plot_data = df.groupby(by="Mentions co-creation with end-users").count()["ID"].rename("Count")
plot_data.index = plot_data.index.map({1: "yes", 0: "no"})
draw_barplot(plot_data, "Mentions co-creation with end-users", (6, 1))
```

In [24]:

```
plot_data = df[df["Mentions co-creation with end-users"]==1].groupby(by="Type of end-user")["ID"].count().rename("")
draw_barplot(plot_data, "Mentioned end-users for co-creation", (5,1.3))
```

# Results overview¶

In [25]:

```
def wrap_label(label, max_length=20):
    if len(label) > max_length:
        space_index = label.find(' ', max_length)
        if space_index != -1:
            return label[:space_index] + '\n' + label[space_index + 1:]
    return label

plot_data_countries = df["Country"].str.get_dummies(sep=';').sum(min_count=1).sort_index(ascending=True).sort_values(ascending=False, kind='mergesort')
series_list = [plot_data_countries, plot_data_biomes.sort_values(ascending=False), plot_data_digital_systems.sort_values(ascending=False), plot_data_taxonomic_classes.sort_values(ascending=False), plot_data_intended_uses.sort_values(ascending=False), plot_data_end_users.sort_values(ascending=False)]
titles = ["1. Countries", "2. Biomes", "3. Digital Systems", "4. Taxonomic Classes", "5. Intended Uses", "6. End-user Types"]
thresholds = [1,3,2.2,2,3,2]
fig, axs = plt.subplots(3, 2, figsize=(20, 23))
axs = axs.flatten()
for ax, series, title, threshold in zip(axs, series_list, titles, thresholds):
    percentages = series / series.sum() * 100
    labels = [f"{name.replace(",", ",\n")} ({value})" if percent >= threshold else "" for name, value, percent in zip(series.index, series, percentages)]
    labels = [wrap_label(label).replace(" Of ", "\nof ") for label in labels]
    ax.pie(series, labels=labels, autopct=lambda p: f"{p:.1f}%" if p >= threshold else "", startangle=90, pctdistance=0.8, labeldistance=1.1, textprops={'fontsize': 15}, counterclock=False)
    ax.axis("equal")
    ax.set_title(title, fontsize=20, pad=20)

fig.subplots_adjust(hspace=0.3)
fig.subplots_adjust(wspace=0.3)
plt.show()
```

# Top scorers¶

In [26]:

```
percentage = 1
threshold = df[score_total].quantile(1-percentage/100)
top = df[df[score_total] >= threshold]
top = top[["Title", "DOI", "Type", "Year", "Abstract", "Parameters", "Feedbacks", "Design", "Intent", "Explains LP interactions", "Visualizations", "Statistical modelling", "Machine Learning", "Data Storing & Sharing", "Mentions transformational potential", score1, score2, score_total]].fillna(0)
top[["Parameters", "Feedbacks", "Design", "Intent", "Explains LP interactions", "Visualizations", "Statistical modelling", "Machine Learning", "Data Storing & Sharing", "Mentions transformational potential", score1, score2]] = top[["Parameters", "Feedbacks", "Design", "Intent", "Explains LP interactions", "Visualizations", "Statistical modelling", "Machine Learning", "Data Storing & Sharing", "Mentions transformational potential", score1, score2]].apply(pandas.to_numeric, downcast='integer')
top
```

Out[26]:

|  | Title | DOI | Type | Year | Abstract | Parameters | Feedbacks | Design | Intent | Explains LP interactions | Visualizations | Statistical modelling | Machine Learning | Data Storing & Sharing | Mentions transformational potential | Systemic Depth | Digital Maturity | Transformative Potential |
| --- | --- | --- | --- | --- | --- | --- | --- | --- | --- | --- | --- | --- | --- | --- | --- | --- | --- | --- |
| 188 | Evaluating the efficacy of a landscape scale f... | 10.1038/s41598-018-23495-z | Article | 2018 | The impact of introduced predators is a major ... | 2 | 2 | 2 | 0 | 0 | 1 | 4 | 0 | 0 | 0 | 9 | 5 | 6.708204 |
| 309 | Mitigating human-wildlife conflict and monitor... | 10.1093/biosci/biad076 | Article | 2023 | The recovery of wild tigers in India and Nepal... | 2 | 2 | 2 | 1 | 0 | 3 | 1 | 3 | 0 | 1 | 10 | 7 | 8.366600 |
| 365 | Acoustic Monitoring Confirms Significant Poach... | 10.3390/ani13040687 | Article | 2023 | Simple Summary The European turtle dove (Strep... | 2 | 2 | 2 | 0 | 1 | 1 | 2 | 2 | 0 | 1 | 11 | 5 | 7.416198 |
| 691 | Effects of Vessel Distance and Sex on the Beha... | 10.3389/fmars.2020.582182 | Article | 2021 | Accurate knowledge of behavior is necessary to... | 2 | 2 | 2 | 0 | 1 | 3 | 3 | 0 | 2 | 1 | 11 | 8 | 9.380832 |
| 884 | Dark roads aid movement but increase mortality... | 10.1002/ecs2.4508 | Article | 2023 | Road networks pose many well-documented threat... | 2 | 2 | 2 | 0 | 0 | 2 | 3 | 0 | 2 | 0 | 9 | 7 | 7.937254 |
| 928 | Citizen science and machine learning: Interdis... | 10.3389/fmars.2022.961095 | Article | 2022 | Successful conservation efforts often require ... | 2 | 2 | 2 | 0 | 1 | 2 | 1 | 3 | 0 | 1 | 11 | 6 | 8.124038 |

# Identification of structural factors¶

In [27]:

```
import statsmodels.api as sm
from statsmodels.robust.robust_linear_model import RLM
```

### Type of end-user¶

In [28]:

```
plot_data = df.copy()
plot_data["Type of end-user"] = plot_data["Type of end-user"].fillna("")
plot_data["Type of end-user"] = plot_data["Type of end-user"].apply(lambda val: "[Not specified]" if val == "" else ("Professionals (Conservationist, Ecologist, Wildlife Manager)" if (val == "Wildlife Manager" or val == "Conservationist" or val == "Ecologist") else "Involving community (Citizen, Farmer, Forester)"))
agg_funcs = {"Type of end-user": ["count"], "Systemic Depth": ["mean"],"Digital Maturity": ["mean"],"Transformative Potential": ["mean"]}
groups = (plot_data.groupby("Type of end-user").agg(agg_funcs).reset_index())
groups.columns = ["Team composition", "Count", "Mean Systemic Depth", "Mean Digital Maturity", "Mean Transformative Potential"]
groups
```

Out[28]:

|  | Team composition | Count | Mean Systemic Depth | Mean Digital Maturity | Mean Transformative Potential |
| --- | --- | --- | --- | --- | --- |
| 0 | Involving community (Citizen, Farmer, Forester) | 33 | 5.151515 | 3.212121 | 3.812238 |
| 1 | Professionals (Conservationist, Ecologist, Wil... | 346 | 3.369942 | 3.982659 | 3.387179 |
| 2 | [Not specified] | 145 | 2.441379 | 3.503448 | 2.719861 |

#### Involving professionals¶

In [29]:

```
regression_data = plot_data[["Type of end-user", "Transformative Potential"]].copy()
regression_data = regression_data.loc[regression_data["Type of end-user"] != "Involving community (Citizen, Farmer, Forester)"]
regression_data["Professionals (Conservationist, Ecologist, Wildlife Manager)"] = regression_data["Type of end-user"].map({"[Not specified]":0, "Professionals (Conservationist, Ecologist, Wildlife Manager)":1})
print(RLM(regression_data["Transformative Potential"], sm.add_constant(regression_data["Professionals (Conservationist, Ecologist, Wildlife Manager)"]), M=sm.robust.norms.HuberT()).fit().summary())
```

```
                       Robust linear Model Regression Results                       
====================================================================================
Dep. Variable:     Transformative Potential   No. Observations:                  491
Model:                                  RLM   Df Residuals:                      489
Method:                                IRLS   Df Model:                            1
Norm:                                HuberT                                         
Scale Est.:                             mad                                         
Cov Type:                                H1                                         
Date:                      Tue, 06 Jan 2026                                         
Time:                              15:25:29                                         
No. Iterations:                          11                                         
================================================================================================================================
                                                                   coef    std err          z      P>|z|      [0.025      0.975]
--------------------------------------------------------------------------------------------------------------------------------
const                                                            2.6181      0.083     31.711      0.000       2.456       2.780
Professionals (Conservationist, Ecologist, Wildlife Manager)     0.6938      0.098      7.055      0.000       0.501       0.887
================================================================================================================================

If the model instance has been used for another fit with different fit parameters, then the fit options might not be the correct ones anymore .
```

#### Involving community¶

In [30]:

```
regression_data = plot_data[["Type of end-user", "Transformative Potential"]].copy()
regression_data = regression_data.loc[regression_data["Type of end-user"] != "Professionals (Conservationist, Ecologist, Wildlife Manager)"]
regression_data["Involving community (Citizen, Farmer, Forester)"] = regression_data["Type of end-user"].map({"[Not specified]":0, "Involving community (Citizen, Farmer, Forester)":1})
print(RLM(regression_data["Transformative Potential"], sm.add_constant(regression_data["Involving community (Citizen, Farmer, Forester)"]), M=sm.robust.norms.HuberT()).fit().summary())
```

```
                       Robust linear Model Regression Results                       
====================================================================================
Dep. Variable:     Transformative Potential   No. Observations:                  178
Model:                                  RLM   Df Residuals:                      176
Method:                                IRLS   Df Model:                            1
Norm:                                HuberT                                         
Scale Est.:                             mad                                         
Cov Type:                                H1                                         
Date:                      Tue, 06 Jan 2026                                         
Time:                              15:25:29                                         
No. Iterations:                          24                                         
===================================================================================================================
                                                      coef    std err          z      P>|z|      [0.025      0.975]
-------------------------------------------------------------------------------------------------------------------
const                                               2.6077      0.081     32.182      0.000       2.449       2.767
Involving community (Citizen, Farmer, Forester)     1.0274      0.188      5.460      0.000       0.659       1.396
===================================================================================================================

If the model instance has been used for another fit with different fit parameters, then the fit options might not be the correct ones anymore .
```

### Co-creation¶

In [31]:

```
plot_data = df.copy()
agg_funcs = {"Mentions co-creation with end-users": ["count"], "Systemic Depth": ["mean"],"Digital Maturity": ["mean"],"Transformative Potential": ["mean"]}
plot_data["Mentions co-creation with end-users"] = plot_data["Mentions co-creation with end-users"].apply(lambda val: "yes" if val == 1 else "no")
groups = (plot_data.groupby("Mentions co-creation with end-users").agg(agg_funcs).reset_index())
groups.columns = ["Mentions co-creation with end-users", "Count", "Mean Systemic Depth", "Mean Digital Maturity", "Mean Transformative Potential"]
groups
```

Out[31]:

|  | Mentions co-creation with end-users | Count | Mean Systemic Depth | Mean Digital Maturity | Mean Transformative Potential |
| --- | --- | --- | --- | --- | --- |
| 0 | no | 497 | 3.140845 | 3.810865 | 3.193025 |
| 1 | yes | 27 | 4.777778 | 3.629630 | 3.896820 |

In [32]:

```
regression_data = plot_data[["Mentions co-creation with end-users", "Transformative Potential"]].copy()
regression_data["Mentions co-creation with end-users"] = regression_data["Mentions co-creation with end-users"].map({"no": 0, "yes": 1})
print(RLM(regression_data["Transformative Potential"], sm.add_constant(regression_data["Mentions co-creation with end-users"]), M=sm.robust.norms.HuberT()).fit().summary())
```

```
                       Robust linear Model Regression Results                       
====================================================================================
Dep. Variable:     Transformative Potential   No. Observations:                  524
Model:                                  RLM   Df Residuals:                      522
Method:                                IRLS   Df Model:                            1
Norm:                                HuberT                                         
Scale Est.:                             mad                                         
Cov Type:                                H1                                         
Date:                      Tue, 06 Jan 2026                                         
Time:                              15:25:29                                         
No. Iterations:                          19                                         
=======================================================================================================
                                          coef    std err          z      P>|z|      [0.025      0.975]
-------------------------------------------------------------------------------------------------------
const                                   3.1130      0.050     62.632      0.000       3.016       3.210
Mentions co-creation with end-users     0.5719      0.219      2.612      0.009       0.143       1.001
=======================================================================================================

If the model instance has been used for another fit with different fit parameters, then the fit options might not be the correct ones anymore .
```

### Using multiple digital systems¶

In [33]:

```
plot_data = df.copy()
plot_data["Used digital systems"] = plot_data["Used digital systems"].fillna("")
plot_data["Number of digital systems"] = plot_data["Used digital systems"].apply(lambda x: len(x.split(';')))
agg_funcs = {"Number of digital systems": ["count"], "Systemic Depth": ["mean"],"Digital Maturity": ["mean"],"Transformative Potential": ["mean"]}
groups = (plot_data.groupby("Number of digital systems").agg(agg_funcs).reset_index())
groups.columns = ["Number of digital systems", "Count", "Mean Systemic Depth", "Mean Digital Maturity", "Mean Transformative Potential"]
groups
```

Out[33]:

|  | Number of digital systems | Count | Mean Systemic Depth | Mean Digital Maturity | Mean Transformative Potential |
| --- | --- | --- | --- | --- | --- |
| 0 | 1 | 365 | 3.076712 | 3.824658 | 3.161550 |
| 1 | 2 | 125 | 3.360000 | 3.832000 | 3.318963 |
| 2 | 3 | 34 | 4.323529 | 3.441176 | 3.626803 |

In [34]:

```
regression_data = plot_data[["Number of digital systems", "Transformative Potential"]].copy()
print(RLM(regression_data["Transformative Potential"], sm.add_constant(regression_data["Number of digital systems"]), M=sm.robust.norms.HuberT()).fit().summary())
```

```
                       Robust linear Model Regression Results                       
====================================================================================
Dep. Variable:     Transformative Potential   No. Observations:                  524
Model:                                  RLM   Df Residuals:                      522
Method:                                IRLS   Df Model:                            1
Norm:                                HuberT                                         
Scale Est.:                             mad                                         
Cov Type:                                H1                                         
Date:                      Tue, 06 Jan 2026                                         
Time:                              15:25:29                                         
No. Iterations:                          15                                         
=============================================================================================
                                coef    std err          z      P>|z|      [0.025      0.975]
---------------------------------------------------------------------------------------------
const                         2.8740      0.118     24.328      0.000       2.642       3.106
Number of digital systems     0.1910      0.079      2.417      0.016       0.036       0.346
=============================================================================================

If the model instance has been used for another fit with different fit parameters, then the fit options might not be the correct ones anymore .
```

### Influencing human behaviour¶

In [58]:

```
plot_data = df.copy()
plot_data["Intended uses"] = plot_data["Intended uses"].fillna("")
plot_data["Influencing humans"] = plot_data["Intended uses"].apply(lambda val: "Influencing humans" if "Influencing Human Behaviour" in val else "Not influencing humans")
agg_funcs = {"Intended uses": ["count"], "Systemic Depth": ["mean"],"Digital Maturity": ["mean"],"Transformative Potential": ["mean"]}
groups = (plot_data.groupby("Influencing humans").agg(agg_funcs).reset_index())
groups.columns = ["Intended uses", "Count", "Mean Systemic Depth", "Mean Digital Maturity", "Mean Transformative Potential"]
groups
```

Out[58]:

|  | Intended uses | Count | Mean Systemic Depth | Mean Digital Maturity | Mean Transformative Potential |
| --- | --- | --- | --- | --- | --- |
| 0 | Influencing humans | 42 | 6.428571 | 3.333333 | 4.336651 |
| 1 | Not influencing humans | 482 | 2.946058 | 3.842324 | 3.132797 |

In [59]:

```
regression_data = plot_data[["Influencing humans", "Transformative Potential"]].copy()
regression_data["Influencing humans"] = regression_data["Influencing humans"].map({"Not influencing humans":0, "Influencing humans":1})
print(RLM(regression_data["Transformative Potential"], sm.add_constant(regression_data["Influencing humans"]), M=sm.robust.norms.HuberT()).fit().summary())
```

```
                       Robust linear Model Regression Results                       
====================================================================================
Dep. Variable:     Transformative Potential   No. Observations:                  524
Model:                                  RLM   Df Residuals:                      522
Method:                                IRLS   Df Model:                            1
Norm:                                HuberT                                         
Scale Est.:                             mad                                         
Cov Type:                                H1                                         
Date:                      Mon, 05 Jan 2026                                         
Time:                              17:17:50                                         
No. Iterations:                          21                                         
======================================================================================
                         coef    std err          z      P>|z|      [0.025      0.975]
--------------------------------------------------------------------------------------
const                  3.0403      0.048     63.586      0.000       2.947       3.134
Influencing humans     1.1064      0.169      6.551      0.000       0.775       1.437
======================================================================================

If the model instance has been used for another fit with different fit parameters, then the fit options might not be the correct ones anymore .
```
